# Parental Stress and Well-Being: A Meta-analysis

**DOI:** 10.1007/s10567-025-00515-9

**Published:** 2025-03-08

**Authors:** Petruța P. Rusu, Octav-Sorin Candel, Ionela Bogdan, Cornelia Ilciuc, Andreea Ursu, Ioana R. Podina

**Affiliations:** 1 Department of Educational Sciences, University Ștefan cel Mare of Suceava, Suceava, Romania; 2 Departement of Psychology, Faculty of Psychology and Educational Sciences, Alexandru Ioan Cuza University of Iași, Iași, Romania; 3 Laboratory of Cognitive Clinical Sciences, University of Bucharest, Bucharest, Romania

**Keywords:** Parental stress, Well-being, Children, Quality analysis, Parental Stress Index

## Abstract

The current meta-analysis aims to investigate the relationship between parental stress and well-being, by focusing on the positive dimensions of parental well-being, such as happiness and life satisfaction, which are often overlooked in favor of negative outcomes like depression. It also extends the scope beyond specific populations, such as parents of children with special needs, to include parents of typically developing children. This meta-analysis included evidence from cross-sectional, longitudinal, daily diary, and intervention studies. By examining various moderators related to parent and child variables (such as gender, age, job status) and study characteristics, this analysis aims to inform more effective, targeted interventions to enhance parental well-being. Systematic searches of the Web of Science, PubMed, APA PsychNet, Scopus, ProQuest, and Google Scholar databases yielded 86 studies, involving 22,108 parents of children aged 0 to 18 years. The PROSPERO registration number for this study is CRD42023428750. Elected outcomes were continuous self-report measures to assess stress and well-being of parents. Our findings indicated a significant negative medium-sized association between parental stress and well-being (*r* = − .40), i.e., greater parental stress was robustly linked to reduced well-being. Higher-quality studies reported stronger stress-well-being associations, underscoring the role of methodological rigor in producing reliable estimates. Additionally, studies utilizing the Parental Stress Index (PSI) demonstrated stronger associations, highlighting its utility as a validated measure for capturing parental stress. Overall, these findings emphasize the need for evidence-based prevention and intervention programs to address parental stress and improve well-being.

**Supplementary Information:**

The online version contains supplementary material available at 10.1007/s10567-025-00515-9.

## Introduction

Parenting is a challenging and rewarding journey that can bring happiness, sense of purpose, fulfillment, and personal growth. At the same time, parenting is also demanding and often stressful, as parents not only care for their children’s physical and emotional needs, but also deal with the pressure of work and life in general.

Parental stress refers to the emotional, psychological, and physical strain experienced by parents in response to various challenges and demands associated with parenting. It encompasses feelings of being overwhelmed, anxious, frustrated, and exhausted, often resulting from factors such as parenting responsibilities, financial pressures, marital conflicts, lack of social support, work-life balance, and problems related to the children (Coşkuner Aktaş & Çiçek, 2023; Cusinato et al., 2020; Eek & Axmon, 2013; Friedline et al., 2021; Karimi et al., 2011). The types of parental stress measured in existing studies are presented in Table 1, which aims to provide an overview of the key stress domains assessed in the literature (the list is representative and not exhaustive).

**Table 1:** Terminologies used in existing studies for parental stress and parental well-being

Concept	Terminologies
Parental stress	Parenting stress, mother stress, father stress, family stress, financial stress, work stress, psychosocial strain, role strain, family strain, work–family conflict, parents role strain, emotional strain, daily hassles
Well-being	Emotional well-being, existential well-being, psychological well-being, quality of life, positive emotions, positive affect, life satisfaction, happiness, psychological health, quality of family life, maternal well-being, family well-being, parental happiness

Research conducted in multiple countries and cultures has consistently shown a negative association between parental stress and well-being (Barnett & Gareis, 2006; Bekker & Zijlstra, 1997; Gómez-Ortiz et al., 2023; Kózka & Przybyła-Basista, 2016; Zarit et al., 2009; Zeng et al., 2020). Well-being is a multi-dimensional construct characterized by *emotional well-being*—the presence of positive emotions (i.e., joy, contentment, confidence, engagement), the balance between positive and negative emotions and social-emotional competence (i.e., emotional stability, resilience, empathy), *cognitive well-being*—positive evaluation of oneself, one’s past life, present life, and positive expectations for the future (self-acceptance, autonomy, competence, clear thinking, optimism, meaning in life, life satisfaction and environmental mastery, satisfaction with specific domains such as marital satisfaction, parental well-being, job satisfaction), and *positive functioning* (i.e., vitality, engagement, positive relationships, personal growth) (Diener, 1984; Diener et al., 2017; Huppert, 2009; Luhmann et al., 2012; Marsh et al., 2020; Ryff, 1989; Watcharakitippong et al., 2017). According to the World Health Organization (WHO, 2004) well-being is a state that allows people to achieve their potential, manage life’s stresses, work productively, and contribute to their community, highlighting the crucial role of mental health in individual and societal well-being. Also, researchers distinguished between positive aspects of well-being (e.g., positive emotions, life satisfaction), and negative aspects of well-being (e.g., negative emotions, depression, anxiety) (Huppert & Whittington, 2003; Karademas, 2007). The present meta-analysis will focus on positive well-being (see Table 1).

Existing research has demonstrated that parental stress is negatively associated with positive indicators of well-being, such as quality of life (Chu et al., 2022; Dardas & Ahmad, 2015; Droogmans et al., 2021), life satisfaction (Dellve et al., 2006; Lubiewska & Derbis, 2016), and happiness (Aramburu et al., 2022; Findler et al., 2016). Parental stress also adversely impacts couple dynamics, reducing communication quality and weakening the couple relationship (Norlin & Broberg, 2013; Priego-Ojeda & Rusu, 2023; Rusu et al., 2020; Tavares et al., 2019). Furthermore, parental stress increases the risk of internalizing and externalizing behaviors in children (Beckmeyer et al., 2022; Yoon et al., 2015) and has a negative effect on children’s academic performance (Tan et al., 2017), as well as a spillover effect on children’s own levels of stress (Pereira et al., 2023) and negative mindset (Pasarelu et al., 2017; Podina et al., 2013).

Despite the substantial body of research exploring parental stress and its impact on well-being, several critical gaps remain in the literature, particularly regarding a comprehensive meta-analysis focused on parental stress and its associations with the positive dimensions of well-being in the general population. First, the existing research is dominated by reviews rather than meta-analyses, with the latter being relatively scarce. Second, most existing reviews focus on child well-being rather than parental well-being (Jones et al., 2021; Ward & Lee, 2020). Third, prior studies have primarily explored broad aspects of parenting, such as the general relationship between parenthood and well-being (Nomaguchi & Milkie, 2020), without delving into the specific, nuanced effects of parental stress on well-being. While these studies offer a valuable overview, they often fail to consider the detailed associations between stress and various facets of parental well-being. Fourth, reviews and meta-analyses that have addressed parental well-being have largely concentrated on negative aspects of well-being, such as depression (Fang et al., 2021, 2022). While this focus has provided insights into the negative components of well-being related to stress, it does not capture the full spectrum of well-being, especially the positive dimensions such as happiness, quality of life, and positive emotions, which are crucial for understanding the overall well-being of parents. Fifth, much of the existing research has concentrated on parents of children with special needs or parents of preterm infants, who face unique stressors (Cachia et al., 2016; Caporali et al., 2020; Cheng & Lai, 2023; Lee, 2013). This focus leaves a significant gap in understanding how parental stress affects parents of typically developing children, particularly in relation to positive aspects of well-being. Additionally, recent meta-analyses have primarily focused on evaluating the effectiveness of intervention programs aimed at reducing parental stress and enhancing well-being (Eira Nunes et al., 2021; Spencer et al., 2020). While these interventions are essential, they often overlook the need for a comprehensive understanding of the baseline relationship between parental stress and well-being. Without thoroughly examining how stress naturally impacts well-being across diverse family structures and contexts, interventions may lack the necessary precision and fail to address the most pertinent aspects of parental stress. Furthermore, key variables such as parental gender, age, and geographical regions—which significantly influence how stress is experienced and managed—are often underrepresented in the literature. For instance, fathers may experience stress differently than mothers, especially in countries where traditional gender roles are more rigid (Hildingsson & Thomas, 2014). Failing to account for these differences limits the ability to design interventions that are sensitive to the diverse experiences of parents, thus reducing their overall effectiveness.

Therefore, the present meta-analysis seeks to fill these gaps by focusing on the positive aspects of parental well-being, such as happiness, life satisfaction, and quality of life, while exploring how parental stress influences a broader range of family structures and contexts. By addressing these gaps, this meta-analysis will provide a more nuanced understanding of parental stress and well-being, contributing to more effective, targeted interventions for improving parental mental health.

The aim of the present study is to conduct a meta-analysis investigating the association between stress and psychological well-being, while exploring the moderating effects of parent and child characteristics, as well as study-specific factors. The terminologies used in the present meta-analysis for “parental stress” and “well-being” are presented in Table 1. “Parental stress” refers to general stress experienced by parents or specific stress, such as financial and work-related stress within the parenting context. “Psychological Well-being” refers to positive aspects of well-being, including parents’ emotional well-being, positive emotions, quality of life, and happiness.

### Theoretical Models on Parental Stress and Well-Being

The impact of stress on parental well-being is explained in different theoretical frameworks such as the *Stress Process Model* (Pearlin, 1999; Pearlin & Bierman, 2013) and *Demand-Rewards Perspective* (Nelson et al., 2013).

*The Stress Process Model* (SPM, Pearlin, 1999; Pearlin & Bierman, 2013; Pearlin et al., 1981) conceptualizes stress as a multifaceted construct involving three elements—stressors, resources, and stress consequences. *Stressors*, defined as sources of stress, typically manifest in two forms: primary stress produced by major life events and chronic stress (e.g., parental, marital, and professional stress, financial, and time strains). In the same direction, Bodenmann (2005) distinguishes between acute stress (stress experienced in the last seven days) and chronic stress (stress experienced over the last year); internal stress, arising within the couple, and external stress, originating from sources outside the couple, highlighting the directional impact of external stress on internal stress within relationships. *Resources* involve coping, defined as a behavioral or cognitive response to a stressor aimed at preventing or alleviating the damage caused by it (including social support and personal resources; Pearlin, 1999). *Stress outcomes* encompass mental health, physical health, and overall well-being (Pearlin, 1999; Pearlin & Bierman, 2013) with resources serving to buffer or prevent the detrimental effects of stressors on well-being. Thus, individuals with varying levels of exposure to stressors may experience divergent impacts on well-being depending on their available resources (Nomaguchi & Milkie, 2020).

*The Demands-Rewards Perspective* (Nelson et al., 2013) describes parenthood as a mix of demands, and rewards, obstacles and advantages. It acknowledges that parenting entails ongoing physical, mental, and financial exertion, yet it also brings opportunities for personal growth, goal attainment, and heightened self-esteem. Parenthood thus represents a blend of gratifying moments and significant challenges. This viewpoint facilitates the comparison of parents’ experiences with those of non-parents, despite inherent difficulties in data collection. Researchers have utilized this perspective to examine how parenthood influences various aspects of healthy living, including body weight, dietary habits, physical activity, alcohol consumption, and healthcare use. The Demands-Rewards Perspective underscores the paradox of parenthood: while it promotes healthier behaviors and discourages risky habits, the demanding nature of parenting may impede parents’ ability to prioritize self-care.

In this meta-analysis, informed by the *Stress Process Model* and the *Demands-Rewards Perspective* we included studies that measured stress in association with positive indicators of parental well-being. We excluded studies that primarily examined negative dimensions of well-being, such as depression, anxiety, and negative emotions, to better align to the scope of our meta-analysis.

### Moderators of Parental Stress and Well-Being

Previous studies have consistently examined a range of moderating variables that influence the association of stress with well-being. These include *demographic factors* such as age, gender, and socio-economic status (e.g., Cavapozzi et al., 2020; Dyrdal & Lucas, 2013; Wang et al., 2020); *individual characteristics* like coping strategies, emotion regulation, and neuroticism(e.g., Extremera & Rey, 2015; Hutchinson & Williams, 2007; Lee, 2007); *social and relational variables*, including dyadic coping (support partners provide to one another when managing stress) and social support (e.g., Roth et al., 2024; Sharda, 2022); and *contextual factors*, such as cultural and geographical differences (e.g., Ansari et al., 2024). Additionally, meta-analytical research frequently considers study-specific variables, such as the year of publication, the measures used for the primary variables, and overall study quality, as potential moderators to address methodological variability (e.g., Cohen et al., 2024). These factors are critical for understanding variations in research outcomes and ensuring the robustness of meta-analytic findings.

Drawing on this established body of literature and the availability of data within the included studies, the current meta-analysis examined the following moderators: parental gender, age, and education; child gender and age; maternal employment status; child health status; geographical region, relationship status; methods of stress measurement; the year of study publication and the study quality. These variables were selected to account for individual, familial, contextual variations, as well as methodological rigor, ensuring a comprehensive examination of factors moderating the association between parental stress and well-being, while remaining aligned with data availability in the analyzed studies.

#### Parents’ Gender & Age

Existing research showed inconsistent findings in terms of stress and well-being differences in mothers and fathers. Some studies indicated that mothers might experience higher levels of stress than fathers, while others found no differences. A meta-analysis in parenting stress (Pinquart, 2018) that included 457 studies revealed that mothers experience higher stress levels than fathers. The high level of stress in mothers could be attributed to the high amount of multitasking like childcare and housework, high work–family conflict (Offer & Schneider, 2011), and time pressure (Ruppanner et al., 2019). Mothers also report more stress and exhaustion than fathers, especially when partners work long hours (Connelly & Kimmel, 2015; Craig & Brown, 2017; Gómez‐Ortiz et al., 2023; Musick et al., 2016). This discrepancy may arise because mothers tend to fulfill familial responsibilities even while working extensive hours (Nomaguchi & Milkie, 2020). However, recent studies indicated similar stress levels in mothers and fathers (Droogmans et al., 2021; Matalon et al., 2022). There is also evidence that mothers and fathers experience comparable work–family conflicts (Gonçalves et al., 2018) and stress from inflexible jobs (Nomaguchi & Johnson, 2016).

In addition, existing studies found gender disparities in parental well-being. Moreover, the effect of parental stress on well-being is different for mothers and fathers. In the last decades, parenting has undergone a paradigm transformation, especially in what concerns fathers. Although mothers remain the principal caregivers of their children, fathers are more involved in their children’s growth and education (Craig, 2006; Milkie et al., 2002; Yavorsky et al., 2015). Nevertheless, analyzing the answers of 18.000 participants in three different studies, Nelson-Coffey et al. (2019) found that mothers have lower well-being than fathers, even though we refer to overall well-being, subjective well-being (the presence of positive emotions and the absence of negative emotions), or happiness. The explanation for these differences can be attributed to multiple different aspects specific to each parent. While fathers, especially those from western countries (such as the United States) more often engage in leisure activities with children (Musick et al., 2016), mothers around the world spend more time on childcare and housework (Parker & Wang, 2013; Yavorsky et al., 2015), impacting both their well-being and the family’s quality of life as stressed fathers participate less in childcare (Wang et al., 2020). Mothers also showed lower health-related quality of life than fathers (Rohde et al., 2022).

However, the extent to which parents gender moderates the stress–well-being relationship is unclear. When collecting data from both partners it becomes feasible to investigate the effect of one’s parent stress on the other parent’s well-being. Research shows that stressed fathers are less involved in their child care and in consequence this affects not only their own but also mothers’ family quality of life (Wang et al., 2020). The present meta-analysis will further explore whether the association of stress with well-being varies between mothers and fathers.

Although the role of parents’ age has generally been overlooked in research examining the relationship between parental stress and well-being, evidence from a few studies suggests that age could serve as a significant moderator in this relationship. Specifically, the connection between parents’ age and stress appears to be influenced by various life-stage factors. For instance, the sources and intensity of parental stress differ across the lifespan. Younger parents often report higher levels of stress, with a greater number of stressors stemming from family, work, and financial pressures compared to middle-aged parents (Stefaniak et al., 2022). As parents age, the frequency of positive family interactions often increases, potentially lowering stress levels and enhancing overall well-being. Research also highlights specific associations: older parents report fewer marital disagreements but a greater likelihood of shared housework activities between partners (Nomaguchi & Milkie, 2003).

The relationship between parents’ age and well-being presents a complex and mixed picture. Some studies have reported a negative association, such as Aassve et al. (2012), who found that age was negatively related to happiness among both mothers and fathers in a sample of over 9000 parents across 19 European countries. Conversely, other research suggests a positive relationship, indicating that older individuals may experience greater well-being due to a reevaluation of life’s meaning and priorities as they age (Hansen et al., 2009). Furthermore, when the moderating role of parents’ age has been explicitly investigated, findings highlight that age can influence how parenthood impacts life satisfaction. Specifically, older parents tend to experience a more positive reaction to childbirth and adapt more successfully compared to younger parents, with these effects being particularly pronounced among mothers (Dyrdal & Lucas, 2013). Given these mixed findings, this study seeks to examine parents’ age as a potential moderator in the relationship between parental stress and well-being, with the aim of elucidating its complex influences.

#### Parental Education

Parental stress and well-being have been shown to be influenced by education level (Dijkstra-de Neijs et al., 2024; Schieve et al., 2011; Shetty et al., 2024). For instance, a study conducted in the United States found that parents with lower levels of education experienced greater stress related to raising and caring for their children compared to parents with higher education levels (Macomber & Moore, 2016). Additionally, research has indicated that while quality of life tends to improve with higher education, stress levels decrease (Irannejad et al., 2018). However, there is limited understanding of how variations in parental education relate to differences in the stress–well-being relationship. To address this gap, we will examine the moderating role of parental education in the association between stress and well-being.

#### Children’s Gender & Age

The impact of children’s age and gender on parental stress and well-being is complex and not yet fully understood. For instance, studies on parents of children with special needs indicate that gender may play a role in parental well-being. Specifically, mothers of daughters typically report a higher quality of life compared to mothers of sons (Droogmans et al., 2021), whereas parents of boys often experience increased levels of depression, anxiety, and stress (Kumar et al., 2022). In contrast, Barnett and Gareis (2006) observed that parental stress related to after-school activities is significantly more pronounced for parents of girls than for parents of boys, suggesting that the effects of children’ gender on stress and well-being can vary by different parenting contexts.

Conversely, the influence of a child’s age presents a clear pattern: parents of younger children generally report higher well-being and self-esteem, excepting the transition to parenthood, which is marked by a decrease in well-being (Lévesque et al., 2020). As children age and gain independence, parents face increasing challenges, such as academic demands and behavioral changes during adolescence, which contribute to elevated parental stress (Ceballo et al., 2004; Cowan & Cowan, 1992; Nomaguchi, 2012). Given these insights, it is crucial to thoroughly assess how children’s age and gender impact the association of parental stress with well-being. Understanding these dynamics is essential for developing targeted interventions that address the specific needs of parents at different stages of child development, thereby enhancing support mechanisms that are sensitive to the evolving family environment.

#### Mother’s Job Status

Work-related stress significantly impacts parental well-being, primarily through the Work–Family Conflict framework, suggesting that balancing job and family responsibilities challenges many parents, leading to elevated stress and reduced well-being. Specifically, numerous studies have demonstrated the detrimental impact of work-related stress on parental well-being (Ananat & Gassman‐Pines, 2021; Cheng et al., 2021; Leach et al., 2021; Miski Aydin et al., 2023; Wheeler et al., 2011). This strain arises from the conflict between work and family life, leading to Work–Family Conflict or Family–Work Conflict (Fridayanti et al., 2021; Maertz et al., 2019). Work–Family conflict was positively associated with higher levels of stress (Chung et al., 2023; Rabenu et al., 2017) and lower levels of well-being (Ifelunni et al., 2023; Liu et al., 2011; Miller et al., 2022; Neto et al., 2016).

Despite gender equality efforts and increasing workforce participation by mothers, they still disproportionately manage childcare and household responsibilities, heightening their stress and affecting their well-being (Allen et al., 2019; Lachance-Grzela & Bouchard, 2010). A research study across 35 European countries showed that full-time employed mothers experience more inter-role conflicts than their part-time counterparts, further increasing stress (Borgmann et al., 2019). This struggle to meet multiple demands inevitably leads to lower well-being of mothers. Understanding the impact of occupational status on the stress–well-being relationship is crucial. By comparing predominantly employed mothers with unemployed mothers, we can gain insights into how different work situations affect parental well-being.

##### Children’s Health Status

Most of the existing studies investigating parental stress focused on parents of children with special needs (e.g., autism, Down syndrome, genetic disorders, cerebral palsy, Rett syndrome, and intellectual disabilities). Raising a child with special needs brings unique stressors that differ from those experienced by parents of typically developing children. These parents may face emotional challenges, financial strain due to therapy costs and social isolation. Research indicated that parents of children with special needs reported higher levels of grief, anxiety, guilt, shame, depression, and lower levels of well-being than parents of children with typical development (Adams et al., 2018; Findler et al., 2016; Kumar et al., 2022).

The results of a meta-analysis indicated that parents of children with autism might experience higher levels of parenting stress when compared with parents of children with other disabilities and parents of typically developing children (Hayes & Watson, 2013). This might be due to children’s behavioral problems, difficulties in communication and social interaction (Johnson et al., 2011; McKechanie et al., 2017; Pisula & Porębowicz-Dörsmann, 2017; Wang et al., 2020). Managing these behaviors is emotionally and physically challenging for parents. A recent qualitative study found that parents of children with Down syndrome experience the shame-blame complex (the feeling of shame of having a child with cognitive disabilities and the sense of blame associated with having given birth of a child with a detectable condition and blame for inadequacies in parenting) (Scavarda, 2024). For instance, the presence of chronic conditions or disabilities in children not only heightens parents’ emotional distress but may also reduce their coping capacity and access to social support, both of which are essential buffers in the stress–well-being relationship. Consequently, we hypothesize that parents of children with health challenges are likely to exhibit a stronger negative association between stress and well-being due to these compounded psychosocial factors. Therefore, we included children’s health status as a moderator in the analysis of the association between parental stress and well-being.

#### Geographical Region

Regional differences in cultural norms, socio-economic conditions, and access to resources can shape how parents experience and manage stress, ultimately influencing their well-being. Parenting expectations and standards of children’s behavior might differ significantly from one country to another (Markus & Kitayama, 2010; Varnum et al., 2010). Moreover, resource availability, which varies across geographical regions, further underscores the moderating role of the region. For instance, Western nations often provide financial resources, such as public assistance programs or childcare subsidies, which may alleviate financial stress.

In a meta-analysis that investigated the relationship between stress and emotional well-being of medical students, the authors evidenced the moderating role of geographical regions, grouping countries into three groups: East, West, and Middle East (Ansari et al., 2024). In agreement with the approaches and results of previous research, the present study aims to analyze how geographical areas could influence the association between stress and well-being for parents.

#### Relationship Status

The couple relationship can favor the reduction of stress levels and support the well-being of partners through various mechanisms (Bodenmann et al., 2011; DeMaris & Oates, 2022; Pengpid et al., 2024). Within nuclear families, the two parents can share their duties through involvement and financial resources and support each other in stressful situations (Donato et al., 2023; Mangelsdorf et al., 2011). In this way, positive co-parenting and dyadic coping are protective factors for stress management. In the case of single-parent families, the main responsibility for household chores and childcare falls on one parent (Munir et al., 2024), which implies greater efforts and the consumption of many financial, temporal, and energy resources. Previous studies have shown that single mothers experience higher levels of stress and overburden than mothers of nuclear families (Hernández et al., 2009; Olhaberry & Farkas, 2012). Based on these results, we will test the moderating role of relationship status in the relationship between stress and well-being in the case of parents.

### Current Study

The objectives of this meta-analysis are to: (1) systematically evaluate existing research on the relationship between parental stress and well-being, and assess the robustness of this association; (2) explore the role of various moderators on these relationships, including parental and child demographics, health status and geographical area where the studies were conducted. Additionally, this study will consider the type and measurement of stress, as well as the year of study publication, to understand how these factors may influence the observed outcomes. To our best knowledge, this is the first meta-analysis investigating the association between parental stress and well-being.

## Methodology

### Literature Search

**Fig. 1:**
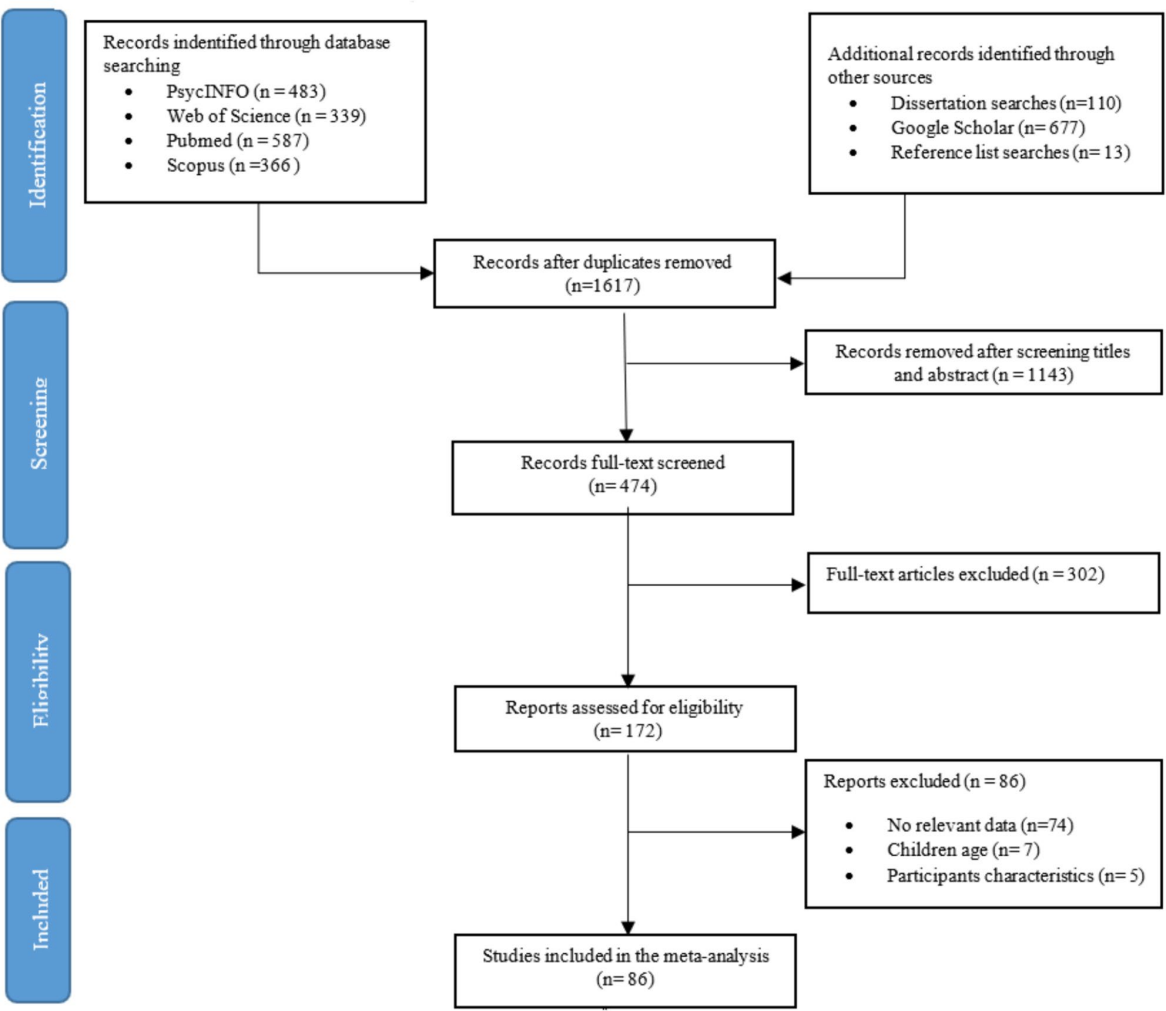
PRISMA flow chart for depicting the studies selection process

This meta-analysis was registered on PROSPERO on the 22nd of May 2023 (record number CRD42023428750). We searched on the following databases (PsycInfo, Scopus, Web of Science, PubMed, and Google Scholar (the first 27 pages of hits searched) to identify potentially relevant studies. To address the gray literature a search on ProQuest Dissertation and Thesis was conducted. The key search terms were (parent* OR mother OR father OR maternal OR paternal OR family) AND (stress OR strain OR hassles) AND (well-being OR wellbeing OR positive emotions OR positive affect OR happy OR happiness OR life satisfaction OR quality of life OR psychological health). The most recent search was completed in September 2024, as outlined in Fig. 1, which illustrates the study selection process, resulting in a total of 86 studies included in the analysis.

### Inclusion/Exclusion Criteria

To be included in the analysis, articles must have: (a) included a continuous self-report measure of the parents’ stress (determined based on whether the studies explicitly identified participants as parents and used stress measures); (b) included a continuous self-report measure of the parents’ well-being; (c) reported the zero-order correlation between parents’ stress and their well-being (if the correlations were not included, standardized regression coefficients were used, when reported); (d) the study included only samples of parents who had minor children (under 18 years old); (e) the article was written in English. We excluded qualitative studies, case reports, protocol studies, editorials, and commentaries. Similarly, studies on parents with adult children were excluded.

### Data Extraction

After completing the comprehensive search, duplicates were removed using Zotero (Ray & Ramesh, 2017), and the remaining titles were assessed. Full texts were then obtained and reviewed for inclusion in the study. Each included study was coded for sample size, mean parents’ and children’ age, parents’ and children’ demographic characteristics (e.g., sex, health status, location, work status of mothers, parents’ education, and relationship status), study method (e.g., cross-sectional, longitudinal). Also, the type of stress and well-being investigated was coded. Next, all the necessary information that would be included in the statistical analyses was double coded by a second reviewer to establish reliability for the variables of interest. Discrepancies were resolved through discussion and ultimately reaching a consensus. In cases where different samples were examined and reported in the same research (for example, the study compared samples from different countries or with different children’ characteristics), each data sample was considered an independent study in our meta‐analysis. However, when the study used dyadic data (and reported correlations for both mothers and fathers), we created an aggregated score for each study. Thus, we tried to maintain the independence of the data. When studies used longitudinal or experimental designs, we extracted data from the baseline measurements.

### Quality Assessment

In order to perform the quality assessment, two authors independently analyzed the extent to which each of the included studies met 5 of the eight criteria proposed by Aromataris and Munn (2020) in the JBI Manual for Evidence Synthesis. Three criteria were eliminated because they do not apply to cross-sectional and longitudinal studies. The five criteria were manually verified for each included study, and they were scored as yes for low risk of bias (green), no for high risk of bias (red), or unclear (yellow) (see Supplementary Materials, Fig. S1). The dissents were examined and decided by consensus. The interrater agreements were 0.81 for the first criterion, 0.64 for the second criterion, 0.86 for the third criterion, 0.93 for the fourth criterion, and 1.00 for the fifth criterion. For each met criteria (yes or low risk of bias), each study received one point, thus obtaining a maximum score of 5. This rating was also used for exploratory testing study quality as a potential moderator for the relationship between parental stress and well-being.

### Statistical Analysis

The study followed the PRISMA guidelines and used the Comprehensive Meta‐Analysis Software (version 4; Borenstein, 2022) to calculate pooled effect sizes. We used the correlation coefficient r as the index of effect size. A pooled effect size (ES) suggests an association between parents’ stress and their well-being. We used Cohen’s (1988) recommendation in regard to the magnitude of effect sizes, namely 0.2 for a small effect, 0.5 for a medium effect, and 0.8 for a large effect. All computations were based on Fisher’s *z* transformation of *r* correlation coefficients from primary studies before the effect sizes were pooled in our meta‐analysis. However, for ease of interpretation, the effect sizes were converted back to the correlation coefficient (*r*). We calculated the pooled ES with and without outliers. An outlier was defined as a study whose 95% CI for the pooled ES was outside the 95% CI for the pooled ES for all studies. For a study to be considered an outlier, there could be no overlap between the 95% CI of that study and the 95% CI of the entire pooled ES.

When more than one effect was offered for the same sample (for example, more than one indicator of well-being, and their relationship with stress, were reported), we randomly selected one of the nonindependent effect sizes to be included for analyses. Effect sizes were extracted from raw correlations, standardized regression coefficients, and sample sizes. When the zero-order correlations were not reported, we transformed the standardized regression coefficients following established guidelines to limit the exclusion of relevant effect sizes (Peterson & Brown, 2005). Finally, sensitivity analyses were performed in order to investigate the robustness of the effect size. To see whether the links between parents’ stress and the various outcomes are different, we examined the associations between parents’ stress and quality of life, health-related quality of life, well-being, emotional well-being, satisfaction with life and psychological problems (Cuijpers, 2016).

For the purposes of the current meta-analysis, we used random effects models, because they allow for wider generalization (Borenstein et al., 2010). For the heterogeneity analysis (how consistent the results were across the analysis), we used the *Q*-tests (which, when significant, indicate heterogeneity in the sample) and *I*^2^ percent, which is a better suited test for larger samples (Higgins, 2003). *I*^2^ takes values from 9 to 100%, with 25%, 50%, and 75% meaning low, medium, and high heterogeneity. To test the effects of various moderators, we used subgroup analyses (with mixed effects models) for the categorical discrete ones, and meta‐regression analyses (with *Z*‐distribution approach) for the continuous ones (Borenstein et al., 2009). In the latter cases, in separate analyses, the correlation effect estimate was regressed on the moderator, which was treated as a covariate. For bias detection, we used a visual inspection of the funnel plot, the Egger *t*-test which, when significant (*p* < .05), indicates bias in the analysis (Egger et al., 1997) and The Duval and Tweedie’s Trim and Fill method which aims at estimating potentially missing studies due to publication bias in the funnel plot and adjusting the overall effect estimate (Shi & Lin, 2019).

## Results

### Main Results

The final sample consisted of 86 studies, with 93 independent samples and a total of 22 1083 parents (see Supplementary Materials, Table S1). Most studies (81) were cross-sectional, while the others were longitudinal (2), daily diary (1) or proposed interventions (2). Nine studies reported dyadic results, for which we aggregated the mothers’ and fathers’ correlations. For most samples (71), the study investigated parental and family stress, while others took into account general stress (15) or other types of stress experienced by parents (Covid-related stress, PTSD, job-related stress; 7). In the first category, most samples (32) measured stress using the Parental Stress Index, in its normal or short forms (Abidin, 1990, 1995). Among the second category, the Perceived Stress Scale (Cohen et al., 1983) was used the most to measure the parents’ general stress (11 studies). When the outcomes are concerned, the most common one was quality of life (25 samples), followed by satisfaction with life (24 samples), well-being (16 samples), health-related quality of life (13 samples each), emotional well-being (10 samples), happiness, flourishing (two samples each), and psychological problems (one sample).

**Fig. 2:**
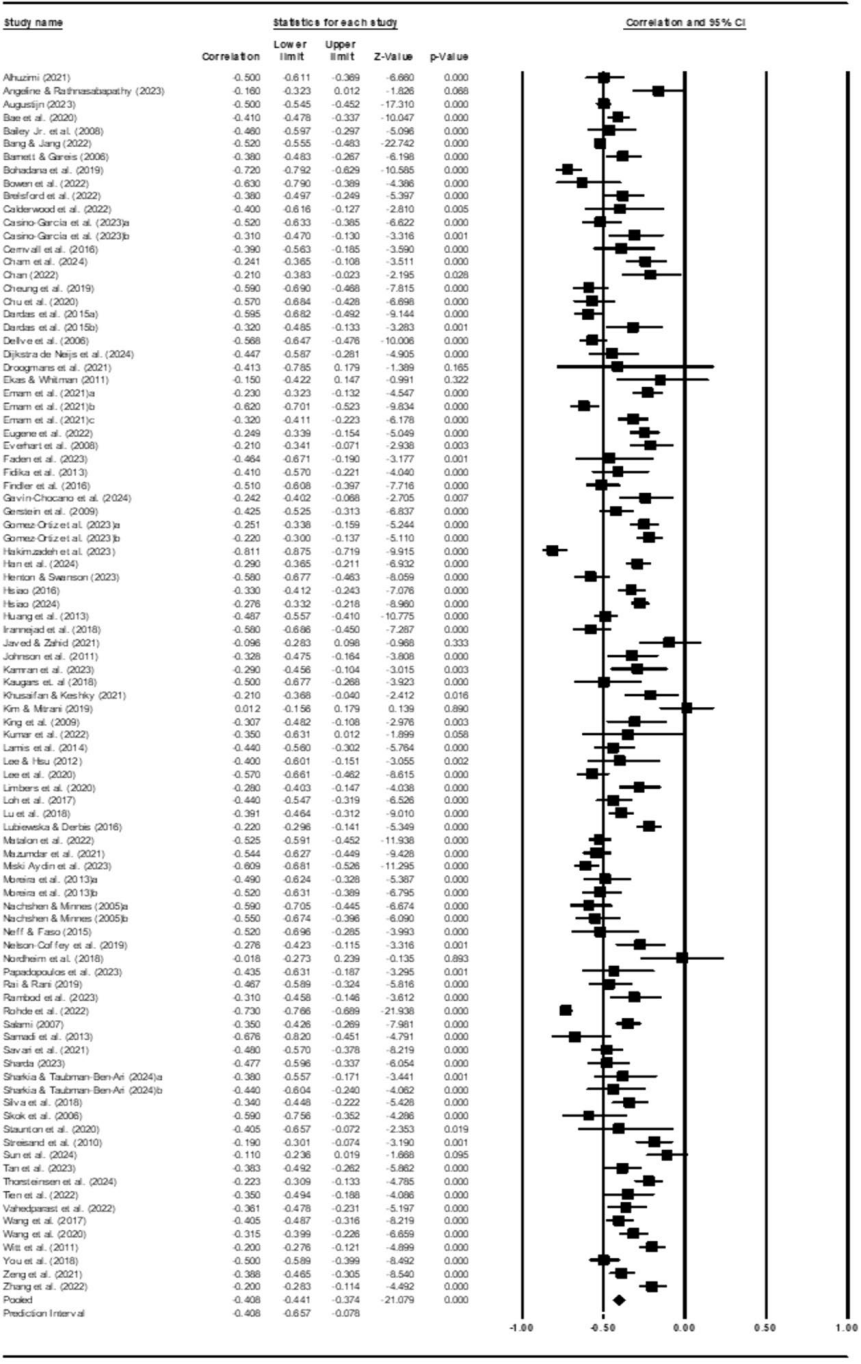
Forest plot displaying estimates and pooled estimates of the effect for the relationship between parents’ stress and their well-being

Firstly, we computed the correlation between the parents’ stress and their levels of well-being. The correlation was significant and had a medium effect size, *r*_*c*_ = − .40, 95% CI [− .44; − .37], *p* < 0.001 (please see Table 2). Figure 2 presents the forest plot describing the pooled effect sizes. The results also indicated significant heterogeneity in the distribution of effect sizes across the included samples (*Q* = 763.84, *p* < .001, *I*^2^ = 87.95%). Excluding 34 outlier studies did not significantly change the effect size (*r*_*c*_ = − .40, 95% CI [− .42; − .37], *p* < 0.001), but reduced the heterogeneity (*Q* = 86.83, *p* = .007, *I*^2^ = 34.35%) (Table 2).

**Table 2:** Overall pooled effect sizes and sensitivity analyses

Pooled effect sizes	Summary information			
	Correlation	95% CI	*p*	*K*
All studies	− .40	[− .44; − .37]	< .001	93
Outliers removed	− .40	[− .42; − .37]	< .001	62
Sensitivity analyses				
Quality of life	− .41	[− .48; − .34]	< .001	25
Health-related quality of life	− .47	[− .55; − .37]	< .001	13
Well-being	− .38	[− .46; − .29]	< .001	16
Emotional well-being	− .40	[− .50; − .28]	< .001	10
Satisfaction with life	− .36	[− .43; − .29]	< .001	24
Happiness	− .49	[− .68; − .24]	< .001	2
Flourishing	− .51	[− .68; − .29]	< .001	2
Psychological problems (SCL-90)	− .42	[− .68; − .06]	< .001	1

We performed a sensitivity analysis to examine the robustness of the results. The associations between parents’ stress and quality of life, health-related quality of life, well-being, emotional well-being, satisfaction with life and psychological problems showed medium effect sizes. The effect sizes for the associations between parents’ stress and happiness, respectively flourishing, were higher. However, the analyses included only two samples/studies (Table 2).

**Fig. 3:**
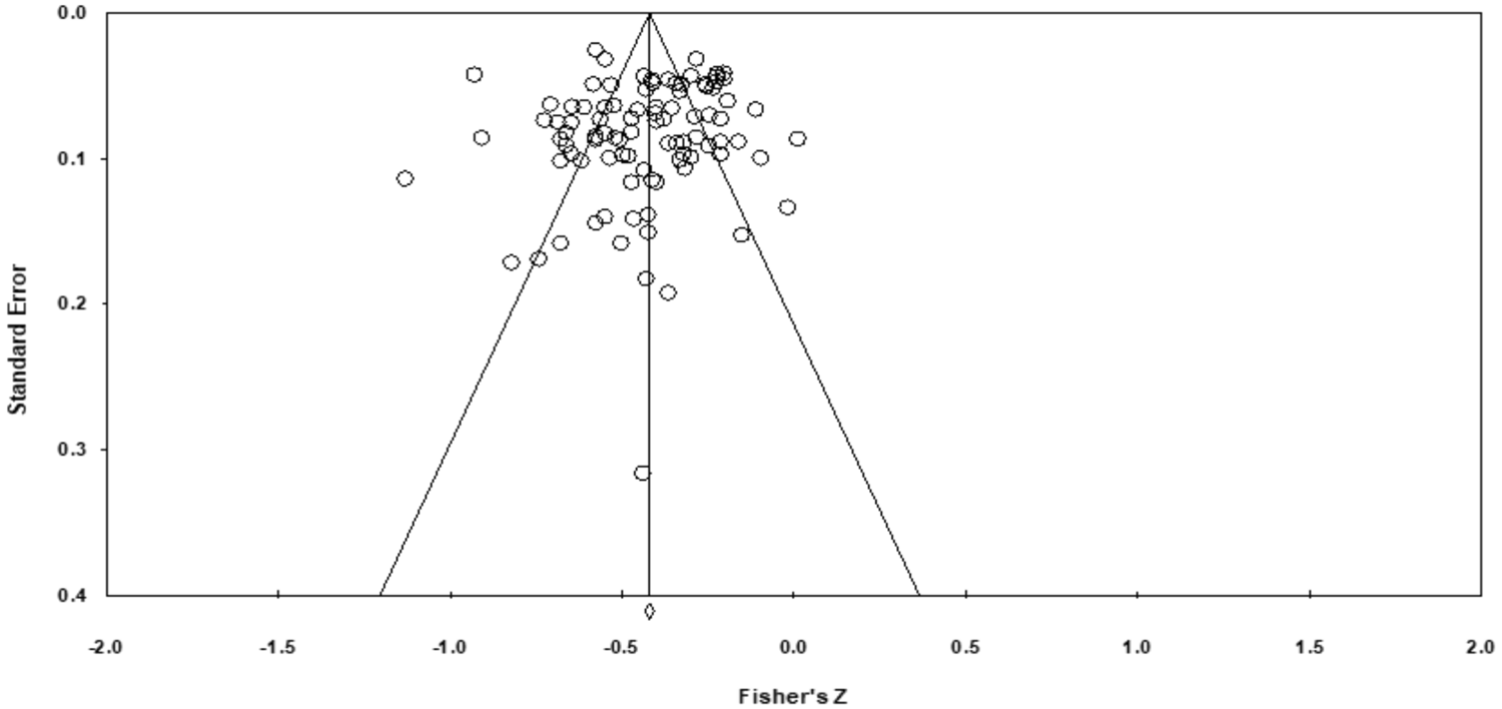
Funnel plot of Standard Error by Fisher’s *Z*

The Egger *t*-test revealed no publication bias, as the test was not significant (intercept = − .47; *p* = .51). The funnel plot, which is presented in Fig. 3, visually confirms this. The Duval and Tweedie’s Trim and Fill method showed that no missing studies can be found, the pooled effect size remaining, thus, unchanged.

Based on the significant heterogeneity of the sample, we computed a series of moderation analyses for the parents’ sample.

### Moderator Analyses

#### Parents and Children’s Gender, Parents and Children’s Age, Mother’s Job Status and Relationship Status, Parents’ Education and Year of Publication

We conducted a series of separate meta-regressions to investigate the role of various potential continuous moderators. In each analysis, the potential moderator was included as a covariate, and the effect estimate was regressed on this variable, resulting in a regression equation for each moderator. For the gender of the parents, we introduced the proportion of female participants (varying from 0 to 100) as a predictor. The results show a non-significant effect of the gender of the parent on the relationship between parents’ stress and their well-being (*b* = − .0003, S.E. = .0009, *p* = 0.75) (see Table 3). Similarly, for the children’s gender, we introduced the proportion of girls as a predictor. The results were also non-significant (*b* = − .0002, S.E. = .001, *p* = 0.90) (Table 3).

**Table 3:** Meta-regression analyses for testing the potential moderating role of parent’s gender, age, education status and relationship status, child’s gender and age, mother’s job status, year of publication, and quality of study

Moderator	*K*	Estimate	S.E.	*z*	*p*
Parent’s gender	83	− .0003	.0009	− 0.31	.75
Parent’s age	67	.002	.004	.41	.67
Child’s gender	46	− .0002	.001	− .12	.90
Child’s age	45	.004	.009	.44	.66
Mother’s job status (proportion of working mothers)	29	− .002	.001	− 1.82	.06
Relationship status (proportion of married/cohabiting parents)	48	− .003	.001	− .18	.85
Parents’ education (proportion of parents who graduated highschool or less)	50	− .0007	.001	− .54	.59
Year of publication	93	− .0001	.0001	− .75	.45
Quality of study	93	− .05	.02	− 1.97	.049

**Fig. 4:**
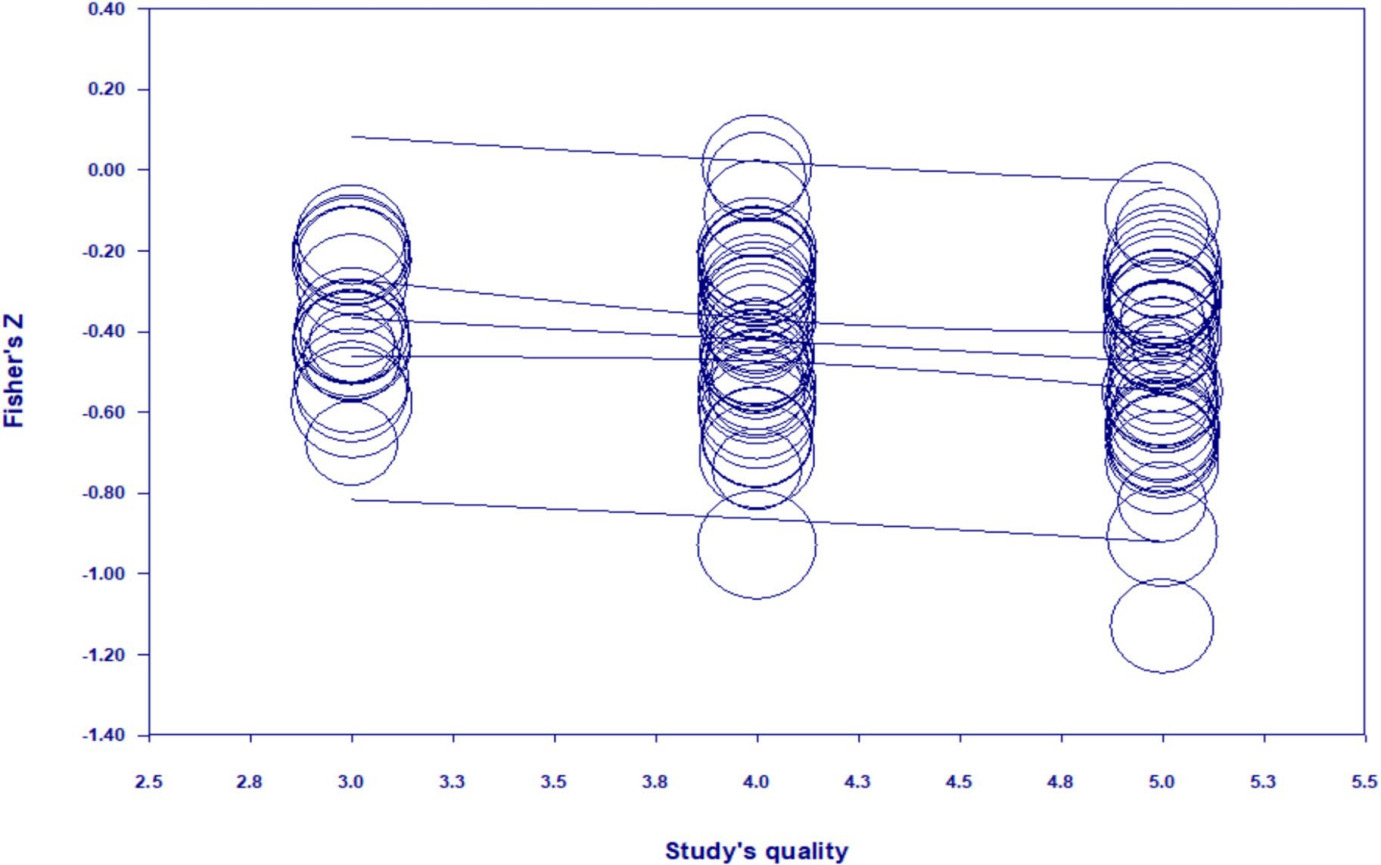
Meta-regression where the effect estimate (the *Y*-axis) was regressed on the study’s quality (on the *X*-axis)

In the third analysis, the age of parents was introduced as a predictor. The results were not significant (*b* = .002, S.E. = .004, *p* = 0.67). The results were also not significant when the age of the children was introduced as a predictor (*b* = .004, S.E. = .009, *p* = 0.66) (Table 3). The studies’ year of publication was also not a significant moderator (*b* = − .0001, S.E. = .0001, *p* = .45). Using the parents’ relationship status (measured as the percentage of parent who were married or cohabited with a partner; *b* = − .003; S.E. = .001, *p* = .85) and their education (measured as the percentage of parents who graduated primary and secondary education; *b* = − .0007; S.E. = .001; *p* = .59) lead to non-significant results. We computed a meta-regression using the proportion of working mothers in each sample (varying from 0 to 100) as a predictor. We excluded from this analysis the samples composed only from fathers for obvious reasons. The results were not significant (*b* = − .002, S.E. = .001, *p* = 0.06). The rating from the quality assessment of each study was, however, a significant moderator (*b* = − .05; S.E. = .02; *p* = 0.049). As the quality of the studies increases, the relationship between stress and well-being becomes more negative (see Fig. 4). Therefore, higher-quality studies tend to report a stronger negative association between stress and well-being.

#### Children’s Health Status, Geographical Area, Type and Measurement of Stress

We used a series of subgroup analyses to test the moderating role of various categorical moderators. The results were not significant when testing for children’s health status (*Q*(3) = 0.36, *p* = .94), the geographical area where the studies were conducted (*Q*(6) = 12.19, *p* = .058), the type of stress assessed by the studies (*Q*(2) = 5.05, *p* = .08). We found, however, significant differences based on the measurement of stress (*Q*(1) = 5.80, *p* = .01). The relationship between stress and well-being was stronger when stress was measured with a version of the Parental Stress Index (*r* = − .46) compared to the cases when it was measured with other scales (*r* = − .37) (See Table 4).

**Table 4:** Subgroup analyses for testing the potential moderating role of children’s health status, geographical area, type of stress and measurement of stress

Moderator	Subgroup summary information				*Q*-test for heterogeneity	
	Correlation	95% CI	*p*	*K*	*Q*	*p*
Children’s health status					.36	.94
Healthy	− .39	[− .45; − .34]	< .001	33		
Mental disability	− .41	[− .47; − .34]	< .001	28		
Physical disability	− .40	[− .47; − .33]	< .001	24		
Various	− .43	[− .53; − .32]	< .001	8		
Geographical area					12.19	.058
Europe	− .41	[− .47; − .34]	< .001	23		
North America	− .33	[− .40; − .26]	< .001	24		
Africa	− .46	[− .65; − .21]	.001	2		
Middle East	− .46	[− .53; − .39]	< .001	19		
South Asia	− .34	[− .48; − .17]	< .001	5		
East Asia	− .40	[− .47; − .33]	< .001	17		
Australia	− .59	[− .72; − .42]	< .001	3		
Type of stress					5.02	.08
Parental and family stress	− .40	[− .44; − .37]	< .001	71		
General stress	− .45	[− .53; − .37]	< .001	15		
Other (PTSD, job-related stress, Covid 19-related stress)	− .28	[− .41; − .15]	< .001	7		
Stress measurement					5.80	.01
Parenting Stress Index	− .46	[− .51; − .40]	< .001	32		
Other	− .37	[− .41; − .33]	< .001	61		

## Discussion

The current meta-analysis investigated the relationship between stress and well-being among parents. Results from pooling 93 independent effect sizes from studies including a total of 22 108 parents revealed a significant negative association of medium magnitude between parental stress and well-being. These findings align with earlier research indicating that parental stress is inversely linked to positive indicators of parental well-being, such as life satisfaction and happiness (Aramburu et al., 2022; Bae et al., 2020; Findler et al., 2016; Lubiewska & Derbis, 2016; Zeng et al., 2020). In contrast to studies that primarily concentrate on negative outcomes such as anxiety, depression, or burnout, this meta-analysis highlights the importance of distinguishing and incorporating positive well-being indicators.

Further analyses explored three types of moderators: parent and child characteristics (such as gender, age, education, relationship status, mother’s job status, children’s health status), contextual factors (i.e., geographical area), and study characteristics (including the type of stress investigated, measures of stress, year of study publication, and study quality). The results showed that parent- and child-related variables, including the gender and age of both parents and children, parental education, maternal employment status, children’s health status, geographical region, and relationship status, did not moderate the association between parental stress and well-being.

While previous studies have reported group-level differences (Pinquart, 2018) and lower well-being of mothers compared to fathers (Nelson-Coffey et al., 2019), our findings suggest that the strength of the relationship between stress and well-being is consistent across genders. This finding aligns with studies demonstrating a negative correlation between parental stress and life satisfaction for both mothers and fathers (Gómez-Ortiz et al., 2023; Matalon et al., 2022) as well as similar levels of work–family conflict reported by both genders (Gonçalves et al., 2018). Moving forward, research efforts should prioritize gathering dyadic data to explore how one parent’s stress impacts not only their own well-being (actor effects) but also the well-being of the other parent (partner effects). Addressing parental stress within relationship education programs that involve both fathers and mothers could prove beneficial, as could integrating considerations of parental stress and well-being into couple and family therapy interventions.

When examining the impact of parents’ age, no moderating effect was observed, indicating that parental stress is consistently linked to lower well-being across different age groups. This is a noteworthy discovery, underscoring the significance of the association between stress and well-being irrespective of parents’ age. This finding contrasts with some previous research suggesting that older parents may show a weaker negative association between stress and well-being compared to younger parents. This could be because older parents often have greater emotional regulation skills, more life experience, and better access to coping resources, allowing them to buffer the effects of stress more effectively (Dyrdal & Lucas, 2013). In contrast, younger parents, who may face more challenges in balancing multiple stressors (e.g., career, finances, and parenting), could exhibit a stronger inverse relationship between stress and well-being. However, the lack of a moderating effect in our analysis implies that age-related factors may not significantly alter the way parental stress influences well-being.

While previous studies reveal the benefits of dual-parenting (Donato et al., 2023; Mangelsdorf et al., 2011) and the downsides of single parenting (Olhaberry & Farkas, 2012), our results showed that the link between parental stress and their well-being remain constant regardless of the parents’ relationship status. Single parenthood, although a potential risk factor for increased stress and lower well-being, can be mitigated by the social support received from other sources, such as the extended family or friends. Also, when interpreting this result, we must take into consideration that most parents included in the study came from married or cohabiting families.

The findings of the current study indicate that parental education did not moderate the association between stress and well-being. While education may influence stress and well-being individually (Irannejad et al., 2018; Macomber & Moore, 2016), it may not significantly alter the strength of their association. This suggests that the link between parental stress and well-being is robust and persists across educational levels. The universal challenges of parenting—such as balancing caregiving demands, managing children’s behavior, and addressing financial pressures—may impact well-being regardless of parents’ educational attainment. Although higher education can provide parents with enhanced coping strategies, access to resources, and advanced problem-solving skills, these advantages may not be sufficient to mitigate the emotional burden of stress when it occurs. Conversely, parents with lower education levels, despite generally reporting higher stress levels, may draw upon alternative support systems or resilience mechanisms that help counterbalance these challenges and reduce disparities. Education may interact with factors such as economic strain, access to childcare, or social support, which were not directly accounted for in the current analysis. Given the critical role of financial strain in shaping individual stress responses and couple dynamics (Falconier et al., 2019; Rusu et al., 2018), future research should examine the interplay between socio-economic factors and stress in predicting well-being across diverse populations.

It is also noteworthy that the relationship between parental stress and well-being was not moderated neither by children’s gender or children’s age. This result contradicts previous studies that emphasize the importance of children’s age and gender in shaping parental experiences (Droogmans et al., 2021; Kumar et al., 2022). Our findings could be explained by the large number of studies included in the present meta-analysis focused on both parents of children with special needs and parents of typically developed children. Another important finding was that the moderation was not significant when considering children’s health status. A high amount of existing studies on stress and well-being focused on parents of children with special needs, as they experience high levels of grief, anxiety, guilt, shame, depression, and lower levels of well-being than parents of children with typical development (Adams et al., 2018; Findler et al., 2016; Kumar et al., 2022). However, our findings suggest that stress is negatively related to well-being also for parents of typically developed children.

The findings from this meta-analysis indicate that the association between parents’ stress and well-being remains consistent across various geographical locations. Another meta-analysis yielded similar results, showing that there were no differences in effect sizes across different geographical regions for psychological factors such as family strengths, positive parenting practices, and family well-being (Dunst, 2021). However, the present study employed moderation analysis by contrasting the effect sizes of studies conducted in North America with those from other regions. The results of this meta-analysis provide a more comprehensive perspective, as we considered more geographical regions such as Europe, North America, Central and South America, Africa, the Middle East, South Asia, East Asia, Australia, and New Zealand.

The type of stress investigated and year of study publication did not moderate the association of parental stress with well-being. However, we found that the association between parental stress and their well-being was stronger when stress was measured using the Parental Stress Index (Abidin, 1990, 1995). The Parental Stress Index is the most widely used instrument in the field and previous research has shown its consistency, reliability, validity, and clinical utility (Holly et al., 2019). Moreover, this measure was validated across multiple settings, clinical groups, and samples (Ríos et al., 2022). Corroborating with past findings, our results show that the Parental Stress Index best captures the difficulties of parenthood and the stress associated with it, making it the most suitable measure to be used when testing the associations with parental well-being.

Finally, we found that study quality was also a significant moderator in the association between stress and well-being, which became stronger as the quality of the studies increased. Our quality analysis assessed studies based on specific criteria, including clearly defined inclusion criteria, detailed descriptions of participants and study settings, the use of objective and standardized measurement tools, valid and reliable outcome measures, and appropriate statistical analyses. Higher-quality studies, which met these criteria more comprehensively, demonstrated a stronger and more consistent association between stress and well-being.

Considering the negative association between parental stress and parents well-being, prevention and intervention programs are needed in order to help parents to cope with stress. These programs might focus on stress management, individual and dyadic coping and parenting education on positive parenting. Couples Coping Enhancement Training, delivered either face-to-face in its traditional format or as an online mobile educational program (CCET, Bodenmann & Shantinath, 2004; Șiean et al., 2024) is particularly relevant in this context, as it focuses on improving partners’ ability to cope with stress together, constructive communication and problem-solving skills. Studies have shown significant positive associations between dyadic coping and both marital satisfaction and individual well-being (Bodenmann et al., 2011; Donato et al., 2023; Randall et al., 2022; Roth et al., 2024; Rusu et al., 2019, 2020). In addition, evidence shows that enhancing dyadic coping skills in CCET is not only linked to improved relationship outcomes, such as greater marital satisfaction, but also to positive parenting behaviors (Zemp et al., 2017).

In addition, access to mental health services for parents is very important. By taking the steps to reduce stress and improve their well-being, parents can create a more positive and supportive environment for themselves and for their children. Family and spousal support serve as valuable resources, as they have been linked to reduced levels of Work–Family Conflict in working parents (Ferri et al., 2018; Landolfi et al., 2020; Minnotte & Minnotte, 2018; Noor et al., 2019). It is crucial to achieve a fair distribution of household chores and caregiving responsibilities between partners, with active involvement from fathers. Research indicates that when both men and women perceive their partners as contributing less to domestic duties, Work–Family Conflict tends to increase (Cerrato & Cifre, 2018). Including dyadic coping strategies in prevention and intervention programs targeted at couples can help raise fathers’ awareness of the importance of supporting their wives and actively participating in household duties.

### Limitations

The current meta-analysis has a number of limitations. First, the heterogeneity in the distribution of effect sizes across the samples might be determined by significant variations of studies in terms of sample characteristics, methodologies and measures of stress and well-being. Also, the high level of heterogeneity also limits the conclusions that can be drawn from this analysis. Second, another limit is related to the design of included studies. Many studies on parental stress and well-being use cross-sectional designs, which capture data at a single point in time. These studies cannot establish causality or track changes over time, limiting the ability to understand the long-term effects of parental stress on well-being. Third, due to our inclusion criteria, we eliminated studies that investigated more complex samples. For example, several studies included both minor children and children over 18 years old, while others also assessed types of caretakers (such as grandparents or siblings). Consequently, we could not include these results in our analyses, as we focused only on parents with minor children.

Finally, interpreting the results from moderators with limited studies per category requires some degree of caution. This is especially true for the geographical area, where certain areas have only two or three studies per category. Similarly, this is also the case of the parenting stress index where there is also an imbalance in between the two categories (32 samples vs. 61). Consequently, these findings should be viewed cautiously before drawing definitive conclusions.

### Future Research

Studies are needed that involve both mothers and fathers and utilize dyadic data analyses to gain a deeper understanding of how the interaction between maternal and paternal stress influences well-being. Examining how maternal stress influences not only her own well-being but also that of the father would enhance our understanding of the dynamics within couples and families, shedding light on how family members mutually influence each other. Additionally, there is a pressing need for more studies focusing on fathers, as mothers are overrepresented in research on stress and well-being. Given the methodological limitations of studies included in the present meta-analysis, future research should adopt longitudinal designs, ecological momentary assessment, and daily diary methods to better grasp how parental stress impacts well-being. Future longitudinal studies will allow a more complex investigation of stress and well-being by considering stress reactivity and stress recovery (Podina et al., 2022).

Since most existing studies rely on self-reported measures, it’s crucial for future investigations to incorporate physiological indicators of acute and chronic stress in parents, such as cortisol levels, heart rate variability, and electroencephalogram readings. Furthermore, delving into mediating mechanisms, such as the quality of the couple relationship, in the link between parental stress and well-being is imperative. There is also a need for studies that address specific chronic stressors experienced by parents, including work-related stress, work–family conflict, goal conflict, couple stress, and the effects of one parent coping with a chronic illness. As our findings indicate a negative relationship between stress and well-being for both parents of children with and without disabilities, conducting more research on parents of typically developing children could provide insights into how various stressors impact family dynamics and parental well-being.

## Conclusion

This meta-analysis revealed a robust negative relationship between parental stress and well-being among parents of minor children, regardless of whether the children had special needs. Notably, significant moderators influenced this association. Specifically, study quality emerged as a key factor, with higher-quality studies reporting a stronger relationship—underscoring the importance of methodological rigor. Additionally, the measure of stress influenced the strength of the association, with studies using the widely validated Parental Stress Index (PSI) demonstrating stronger associations. This highlights the reliability and effectiveness of PSI in capturing the multifaceted nature of parental stress. The findings also suggest that the inverse relationship between parental stress and well-being remains consistent across diverse demographic and contextual factors.

Overall, these findings emphasize the need to address parental stress through evidence-based prevention and intervention programs. Future research should prioritize high-quality study designs, including longitudinal methods, to better understand the dynamics between parental stress and well-being over time. Incorporating validated measures such as the Parental Stress Index (PSI), along with physiological indicators of stress, will enhance the robustness of findings. Additionally, dyadic analyses involving both mothers and fathers are needed to explore how stress impacts not only individual well-being but also broader family systems. By advancing research and implementing targeted interventions, we can better support parents in managing stress, ultimately fostering healthier family environments and improving overall parental well-being.

## Supplementary Information

Below is the link to the electronic supplementary material.

Supplementary file1 (DOCX 25 KB)

Supplementary file2 (DOCX 75 KB)
