# Supplementary material for: Parental Stress and Well-Being: A Meta-analysis
Source: Clin Child Fam Psychol Rev. 2025 Mar 8;28(2):255–74. doi: 10.1007/s10567-025-00515-9 (PMC12162691; doi:10.1007/s10567-025-00515-9)
Supplement: Supplementary file 1 — Supplementary file1 (DOCX 25 KB) [file 10567_2025_515_MOESM1_ESM.docx]

|  | **Inclusion criteria** | **Study subject details** | **Standard criteria for the condition** | **Outcome measurement** | **Statistical analysis** | **Overall study quality (range 0 to 5)** |
| --- | --- | --- | --- | --- | --- | --- |
| **Authors** |  |  |  |  |  |  |
| Alhuzimi (2021) |  |  |  |  |  | **3** |
| Angeline & Rathnasabapathy (2023) |  |  |  |  |  | **3** |
| Augustijn (2023) |  |  |  |  |  | **5** |
| Bae et al. (2020) |  |  |  |  |  | **4** |
| Bailey Jr. et al. (2008) |  |  |  |  |  | **4** |
| Bang & Jang (2022) |  |  |  |  |  | **3** |
| Barnett & Gareis (2006) |  |  |  |  |  | **3** |
| Bohadana et al. (2019) |  |  |  |  |  | **5** |
| Bowen et al. (2022) |  |  |  |  |  | **4** |
| Brelsford et al. (2022) |  |  |  |  |  | **5** |
| Calderwood et al. (2022) |  |  |  |  |  | **4** |
| Casino-García et al. (2023) |  |  |  |  |  | **5** |
| Cernvall et al. (2016) |  |  |  |  |  | **3** |
| Cham et al. (2024) |  |  |  |  |  | **4** |
| Chan (2022) |  |  |  |  |  | **3** |
| Cheung et al. (2019) |  |  |  |  |  | **5** |
| Chu et al. (2020) |  |  |  |  |  | **5** |
| Dardas et al. (2015a) |  |  |  |  |  | **5** |
| Dardas et al. (2015b) |  |  |  |  |  | **4** |
| Dellve et al. (2006) |  |  |  |  |  | **5** |
| Dijkstra‑de Neijs et al. (2024) |  |  |  |  |  | **5** |
| Droogmans et al. (2021) |  |  |  |  |  | **5** |
| Ekas & Whitman (2011) |  |  |  |  |  | **5** |
| Emam et al. (2021) |  |  |  |  |  | **5** |
| Eugene et al. (2022) |  |  |  |  |  | **3** |
| Everhart et al. (2008) |  |  |  |  |  | **5** |
| Faden et al. (2023) |  |  |  |  |  | **4** |
| Fidika et al. (2013) |  |  |  |  |  | **5** |
| Findler et al. (2016) |  |  |  |  |  | **5** |
| Gavín-Chocano et al. (2024) |  |  |  |  |  | **5** |
| Gerstein et al. (2009) |  |  |  |  |  | **4** |
| Gomez-Ortiz et al. (2023) |  |  |  |  |  | **4** |
| Hakimzadeh et al. (2023) |  |  |  |  |  | **5** |
| Han et al. (2024) |  |  |  |  |  | **5** |
| Henton & Swanson (2023) |  |  |  |  |  | **4** |
| Hsiao (2016) |  |  |  |  |  | **4** |
| Hsiao (2024) |  |  |  |  |  | **5** |
| Huang et al. (2013) |  |  |  |  |  | **4** |
| Irannejad et al. (2018) |  |  |  |  |  | **4** |
| Javed & Zahid (2021) |  |  |  |  |  | **4** |
| Johnson et al. (2011) |  |  |  |  |  | **5** |
| Kamran et al. (2023) |  |  |  |  |  | **4** |
| Kaugars et. al (2018) |  |  |  |  |  | **4** |
| Khusaifan & Keshky (2021) |  |  |  |  |  | **4** |
| Kim & Mitrani (2019) |  |  |  |  |  | **4** |
| King et al. (2009) |  |  |  |  |  | **5** |
| Kumar et al. (2022) |  |  |  |  |  | **5** |
| Lamis et al. (2014) |  |  |  |  |  | **4** |
| Lee & Hsu (2012) |  |  |  |  |  | **5** |
| Lee et al. (2020) |  |  |  |  |  | **5** |
| Limbers et al. (2020) |  |  |  |  |  | **4** |
| Loh et al. (2017) |  |  |  |  |  | **4** |
| Lu et al. (2018) |  |  |  |  |  | **5** |
| Lubiewska & Derbis (2016) |  |  |  |  |  | **3** |
| Matalon et al. (2022) |  |  |  |  |  | **4** |
| Mazumdar et al. (2021) |  |  |  |  |  | **4** |
| Miski Aydin et al. (2023) |  |  |  |  |  | **4** |
| Moreira et al. (2013) |  |  |  |  |  | **5** |
| Nachshen & Minnes (2005) |  |  |  |  |  | **5** |
| Neff & Faso (2015) |  |  |  |  |  | **5** |
| Nelson-Coffey et al. (2019) |  |  |  |  |  | **3** |
| Nordheim et al. (2018) |  |  |  |  |  | **4** |
| Papadopoulos et al. (2023) |  |  |  |  |  | **5** |
| Rai & Rani (2019) |  |  |  |  |  | **5** |
| Rambod et al. (2023) |  |  |  |  |  | **5** |
| Rohde et al. (2022) |  |  |  |  |  | **4** |
| Salami (2007) |  |  |  |  |  | **4** |
| Samadi et al. (2013) |  |  |  |  |  | **5** |
| Savari et al. (2021) |  |  |  |  |  | **3** |
| Sharda (2023) |  |  |  |  |  | **4** |
| Sharkia & Taubman-Ben-Ari (2024) |  |  |  |  |  | **4** |
| Silva et al. (2018) |  |  |  |  |  | **5** |
| Skok et al. (2006) |  |  |  |  |  | **3** |
| Staunton et al. (2020) |  |  |  |  |  | **3** |
| Streisand et al. (2010) |  |  |  |  |  | **3** |
| Sun et al. (2024) |  |  |  |  |  | **5** |
| Tan et al. (2023) |  |  |  |  |  | **3** |
| Thorsteinsen et al. (2024) |  |  |  |  |  | **4** |
| Tien et al. (2022) |  |  |  |  |  | **3** |
| Vahedparast et al. (2022) |  |  |  |  |  | **4** |
| Wang et al. (2017) |  |  |  |  |  | **3** |
| Wang et al. (2020) |  |  |  |  |  | **4** |
| Witt et al. (2011) |  |  |  |  |  | **4** |
| You et al. (2018) |  |  |  |  |  | **4** |
| Zeng et al. (2021) |  |  |  |  |  | **5** |
| Zhang et al. (2022) |  |  |  |  |  | **3** |
|  |  |  |  |  |  |  |
| **Interrater agreements** | **0.81** | **0.64** | **0.86** | **0.93** | **1** |  |
