# Supplementary material for: Parental Stress and Well-Being: A Meta-analysis
Source: Clin Child Fam Psychol Rev. 2025 Mar 8;28(2):255–74. doi: 10.1007/s10567-025-00515-9 (PMC12162691; doi:10.1007/s10567-025-00515-9)
Supplement: Supplementary file 2 — Supplementary file2 (DOCX 75 KB) [file 10567_2025_515_MOESM2_ESM.docx]

Table S1. Study characteristics

| **Study** | **N** | **Gender (% of mothers)** | **Parent age** | **Child age** | **Child gender (% of girls)** | **Design** | **Location** | **Type of stress and measure** | **Type of well-being and measure** | **Correlation** | **Regression (Beta)** |
| --- | --- | --- | --- | --- | --- | --- | --- | --- | --- | --- | --- |
| Alhuzimi (2021) | 150 | 74 | No data | No data | 37.3 | Cross-sectional | Middle East | Parental / familial (Parent Stress Index Short Form, Abidin, 1995) | Emotional well-being (12-Item General Health Questionnaire, Goldberg & Williams, 1988) | 0.5 |  |
| Angeline & Rathnasabapathy (2023) | 131 | 100 | No data | No data | 25.2 | Cross-sectional | South Asia | Parental / familial (Parenting Stress Scale, Berry & Jones, 1995) | Well-being (Psychological General Well-Being Index, Grossi et al., 2006) |  | -0.16 |
| Augustijn (2023) | 996 | 100 | 36.9 | 7.7 | No data | Cross-sectional | Europe | General (self-created) | Satisfaction with life (self-created) | -0.5 |  |
| Bae et al. (2020) | 535 | 100 | No data | No data | No data | Cross-sectional | East Asia | Parental / familial (Parenting Stress Index, Abidin, 1986) | Flourishing (PERMA-Profiler, Butler & Kern, 2016) |  | -0.41 |
| Bailey Jr. et al. (2008) | 108 | 100 | 35.3 | 6 | No data | Cross-sectional | North America | Parental / familial (Parenting Stress Index, Abidin, 1986) | Quality of life (The Quality of Life Inventory, Frisch, 1994) | -0.46 |  |
| Bang & Jang (2022) | 1560 | 100 | 36.8 | No data | 48.8 | Cross-sectional | East Asia | Parental / familial (Parenting Stress Scale, Kim & Kang, 1997) | Happiness (Subjective Happiness Scale, Lyubomirsky & Lepper, 1999) | -0.52 |  |
| Barnett & Gareis (2006) | 243 | 83.4 | 39.2 | 9.2 | 50.2 | Cross-sectional | North America | Parental / familial (Parental after-school stress scale, self-created) | Well-being (Affectometer 2, Kammann & Flett, 1983) | -0.38 |  |
| Bohadana et al. (2019) | 139 | 86.33 | 38.85 | 8.9 | 28.8 | Cross-sectional | Australia and New Zeeland | Parental / familial (Parent Stress Index Short Form, PSI-SF, Abidin, 1995) | Quality of life (Quality of Life in Autism Scale, Eapen et al. 2014) | -0.72 |  |
| Bowen et al. (2022) | 38 | 0 | 43 | No data | No data | Cross-sectional | Africa | Parental / familial (Aggravation in Parenting Scale, Ehrle & Moore, 1997) | Flourishing (Secure Flourishing Index, VanderWeele, 2017) | -0.63 |  |
| Brelsford et al. (2022) | 185 | 70.81 | 31.4 | No data | No data | Cross-sectional | North America | Parental / familial (Stanford Acute Stress Reaction Questionnaire, Cardena et al., 2000) | Satisfaction with life (Satisfaction with Life Scale, Diener et al., 1985) | -0.38 |  |
| Calderwood et al. (2022) | 47 | 74.5 | 40.83 | 12.09 | 46.8 | Cross-sectional | North America | Other (job stress, scale developed by Motowidlo et al., 1986) | Emotional well-being (Job-Related Affective Well-Being Scale, Van Katwyk et al., 2000) | -0.4 |  |
| Casino-García et al. (2023) | 135 | 77 | 43.65 | No data | No data | Cross-sectional | Europe | General (Perceived Stress Scale, Cohen et al., 1983) | Emotional well-being (Scale of Positive and Negative Experiences, Diener et al., 2010) |  | -0.52 |
|  | 110 | 81.8 | 44.57 | No data | No data | Cross-sectional | Europe | General (Perceived Stress Scale, Cohen et al., 1983) | Emotional well-being (Scale of Positive and Negative Experiences, Diener et al., 2010) |  | -0.31 |
| Cernvall et al. (2016) | 79 | 69.62 | No data | No data | No data | Cross-sectional | Europe | Other (PTSD, not specified) | Quality of life (not specified) | -0.39 |  |
| Cham et al. (2024) | 207 | 55.6 | 46.47 | No data | No data | Cross-sectional | East Asia | Other (Perceived stress scale modified for COVID-19, Ibrahim et al., 2023) | Satisfaction with life (Satisfaction with Life Scale, Diener et al., 1985) | -0.241 |  |
| Chan (2022) | 109 | 90.8 | 41.22 | 8.98 | 41.3 | Cross-sectional | East Asia | Other (self-created COVID-related stress) | Well-being (World Health Organization Well-Being Index, Psychiatric Research Unit, 1998) | -0.21 |  |
| Cheung et al. (2019) | 136 | 81.62 | 43.09 | 9.39 | 18.3 | Cross-sectional | East Asia | Parental / familial (Parenting Stress Scale, Berry & Jones, 1995) | Well-being (Mental Health Continuum-Short Form, Keyes 2002) | -0.59 |  |
| Chu et al. (2020) | 110 | 72.2 | No data | No data | No data | Cross-sectional | East Asia | Parental / familial (Caregiver Burden Inventory, Novak & Guest, 1989) | Quality of life (CarerQOL, Brouwer et al., 2006) | -0.57 |  |
| Dardas et al. (2015a) | 181 | 62 | 37 | 6.3 | 16 | Cross-sectional | Middle East | Parental / familial (Parent Stress Index Short Form, Abidin, 1995) | Quality of life (World Health Organization Quality of Life Assessment-BREF, WHO, 1996) |  | -0.595 |
| Dardas et al. (2015b) | 101 | 0 | 39.3 | 5.9 | 18.82 | Cross-sectional | Middle East | Parental / familial (Parent Stress Index Short Form, Abidin, 1995) | Quality of life (World Health Organization Quality of Life Assessment-BREF, WHO, 1996) |  | -0.32 |
| Dellve et al. (2006) | 244 | 55.32 | No data | No data | 60.56 | Intervention | Europe | Parental / familial (Swedish Parenthood Stress Questionnaire, Ostberg et al. 1997) | Satisfaction with life (Ladder of Life instrument, Wiklund et al. 1992) |  | -0.568 |
| Dijkstra de Neijs et al. (2024) | 107 | 52.33 | 35.65 | 4.6 | 9.83 | Cross-sectional | Europe | Parental / familial ( Dutch parenting burden questionnaire, Vermulst et al., 2015). | Quality of life (World Health Organization Quality of Life Assessment-BREF, WHO, 1996) |  | -0.447 |
| Droogmans et al. (2021) | 13 | 53.84 | 36.8 | 9.71 | No data | Cross-sectional | Europe | Parental / familial ( Parent Stress Index Short Form, Abidin, 1995) | Quality of life (Family Quality of Life Scale, Hoffman et al., 2006 | -0.413 |  |
| Ekas & Whitman (2011) | 46 | 100 | 40.98 | 10.17 | 21.7 | Daily Diary | North America | General (The Small Life Events Scale, Zautra et al. 1986) | Emotional well-being (The Positive and Negative Affect Schedule, Watson et al. 1988) | -0.15 |  |
| Emam et al. (2021) | 380 | No data | 23.61 | No data | 31.4 | Cross-sectional | Middle East | Parental / familial (Parenting Stress Index, Abidin, 1986) | Satisfaction with life (Satisfaction with Life Scale, Diener et al., 1985) | -0.23 |  |
|  | 187 | No data | 21.44 | No data | 36.4 | Cross-sectional | Middle East | Parental / familial (Parenting Stress Index, Abidin, 1986) | Satisfaction with life (Satisfaction with Life Scale, Diener et al., 1985) | -0.62 |  |
|  | 350 | No data | No data | No data | 40 | Cross-sectional | Middle East | Parental / familial (Parenting Stress Index, Abidin, 1986) | Satisfaction with life (Satisfaction with Life Scale, Diener et al., 1985) | -0.32 |  |
| Eugene et al. (2022) | 397 | 51.88 | 39.74 | 10.93 | 47 | Cross-sectional | North America | Other (Covid-related, Cohen et al., 2020) | Well-being (Warwick-Edinburgh Mental Well-Being Scale, Tennant et al., 2007) | -0.249 |  |
| Everhart et al. (2008) | 193 | 97 | 35.3 | 7.8 | 37 | Cross-sectional | North America | Parental / familial (Family Inventory of Life Events, Patterson & McCubbin, 1983) | Quality of life (Pediatric Asthma Caregiver’s Quality of Life Questionnaire, Juniper et al., 1996) | -0.21 |  |
| Faden et al. (2023) | 43 | 62.79 | No data | No data | No data | Cross-sectional | Middle East | Parental / familial (Parental Stress Scale, Berry & Jones, 1995 | Quality of life (World Health Organization Quality of Life Assessment-BREF, WHO, 1996) | -0.464 |  |
| Fidika et al. (2013) | 89 | 85.6 | 39 | 9 | 51.7 | Cross-sectional | Europe | Parental / familial (Impact on Family Scale, Stein & Riessman, 1980) | Quality of life (The Ulm Quality of Life Inventory for Parents of chronically ill children, Goldbeck & Storck, 2002) |  | -0.41 |
| Findler et al. (2016) | 191 | 100 | 35.79 | 4.77 | No data | Cross-sectional | Middle East | General (Perceived Stress Scale, Cohen et al., 1983) | Happiness (Subjective Happiness Scale, Lyubomirsky & Lepper, 1999) | -0.51 |  |
| Gavín-Chocano et al. (2024) | 123 | 78 | 37.85 | 4.32 | 58.5 | Cross-sectional | Europe | Parental / familial (Parent Stress Index Short Form, Abidin, 1995) | Satisfaction with life (Satisfaction with Life Scale, Diener et al., 1985) | -0.242 |  |
| Gerstein et al. (2009) | 230 | 50 | No data | No data | 33.9 | Longitudinal | North America | Parental / familial (Parenting daily hassles, Crnic & Greenberg, 1990) | Other (Symptom Checklist-35, Derogatis 1993) | 0.425 |  |
| Gomez-Ortiz et al. (2023) | 421 | 78.3 | 38.3 | 9.8 | No data | Cross-sectional | Europe | Parental / familial (Parental Stress Scale, Berry & Jones, 1995 | Satisfaction with life (Satisfaction with Life Scale, Diener et al., 1985) | -0.251 |  |
|  | 525 | 59.9 | 37.08 | 4.66 | No data | Cross-sectional | Europe | Parental / familial (Parental Stress Scale, Berry & Jones, 1995 | Satisfaction with life (Satisfaction with Life Scale, Diener et al., 1985) | -0.22 |  |
| Hakimzadeh et al. (2023) | 80 | 85 | 34.01 | 5.37 | No data | Cross-sectional | Middle East | Parental / familial (Parent Stress Index Short Form, Abidin, 1995) | Health-related quality of life (36‐Item Short Form Health Survey, Ware Jr. et al., 1992) | -0.811 |  |
| Han et al. (2024) | 542 | 50 | 44.13 | No data | 45 | Cross-sectional | East Asia | Parental / familial (Parent Stress Index Short Form, Abidin, 1995) | Satisfaction with life (self-created) | -0.29 |  |
| Henton & Swanson (2023) | 151 | 100 | No data | No data | No data | Cross-sectional | Europe | Parental / familial (Parental Stress Scale, Berry & Jones, 1995 | Well-being (Warwick-Edinburgh Mental Well-Being Scale, Tennant et al., 2007) |  | -0.58 |
| Hsiao (2016) | 429 | No data | No data | No data | No data | Cross-sectional | North America | Parental / familial (Parental Stress Index and the Parental Attitudes about Child rearing Scale, Blumberg et al., 2012) | Quality of life (self-created) | -0.33 |  |
| Hsiao (2024) | 1003 | No data | No data | No data | 19.84 | Cross-sectional | North America | Parental / familial (self-created) | Health-related quality of life (self-created) | -0.276 |  |
| Huang et al. (2013) | 413 | 0 | 37.65 | 4.92 | No data | Cross-sectional | East Asia | Parental / familial (Parental Stress Scale, Berry & Jones, 1995 | Health-related quality of life (36‐Item Short Form Health Survey, Ware Jr. et al., 1992) | -0.487 |  |
| Irannejad et al. (2018) | 124 | No data | 39.63 | 10.38 | 42.4 | Cross-sectional | Middle East | General (Perceived Stress Scale, Cohen et al., 1983) | Health-related quality of life (36‐Item Short Form Health Survey, Ware Jr. et al., 1992) | -0.58 |  |
| Javed & Zahid (2021) | 104 | 100 | No data | No data | No data | Cross-sectional | South Asia | Parental / familial (Kingston Caregiver stress scale, Fatima, 2018) | Satisfaction with life (Satisfaction with Life Scale, Diener et al., 1985) | -0.096 |  |
| Johnson et al. (2011) | 128 | 50 | 40.6 | 7.8 | 19 | Cross-sectional | North America | Parental / familial (Parenting Stress Scale: Autism, Phetrasuwan, 2003) | Health-related quality of life (36‐Item Short Form Health Survey, Ware Jr. et al., 1992) | -0.328 |  |
| Kamran et al. (2023) | 105 | 100 | 31.82 | No data | 61.9 | Cross-sectional | Middle East | Parental / familial (The Parental Stress Scale-Neonatal intensive care unit, Milles et al. 1993) | Health-related quality of life (36‐Item Short Form Health Survey, Ware Jr. et al., 1992) | -0.29 |  |
| Kaugars et. al (2018) | 54 | No data | No data | 7.48 | 14.89 | Cross-sectional | North America | Parental / familial (Parent Stress Index Short Form, Abidin, 1995) | Health-related quality of life (The PedsQL Family Impact Module, Varni et al., 2004) | -0.5 |  |
| Khusaifan & Keshky (2021) | 131 | 55 | No data | No data | 21.4 | Cross-sectional | Middle East | Parental / familial (Family Stress and Coping Interview- Scale, Nachshen et al., 2003) | Satisfaction with life (Satisfaction with Life Scale, Diener et al., 1985) |  | -0.21 |
| Kim & Mitrani (2019) | 137 | 100 | 36.18 | No data | No data | Cross-sectional | North America | Parental / familial (Family hassles scale, Kanner et al., 1981) | Health-related quality of life (PROMIS 29, Cella et al., 2010) | 0.012 |  |
| King et al. (2009) | 91 | 50 | 43.96 | 9.73 | 35 | Cross-sectional | Europe | General (Perceived Stress Scale, Cohen et al., 1983) | Quality of life (World Health Organization Quality of Life Assessment-BREF, WHO, 1996) | -0.307 |  |
| Kumar et al. (2022) | 30 | 86.7 | 32.6 | 9.33 | 20 | Cross-sectional | South Asia | General (Depression, Anxiety and Stress Scale 21, Lovibond & Lovibond, 1995) | Quality of life (World Health Organization Quality of Life Assessment-BREF, WHO, 1996) | -0.35 |  |
| Lamis et al. (2014) | 152 | 100 | 32.33 | No data | No data | Cross-sectional | North America | Parental / familial (Parent Stress Index Short Form, Abidin, 1995) | Well-being (The Spiritual Well-Being Scale, Paloutzian & Ellison, 1991) | -0.44 |  |
| Lee & Hsu (2012) | 55 | 100 | 25.5 | No data | No data | Cross-sectional | North America | General (Perceived Stress Scale, Cohen et al., 1983) | Health-related quality of life (36‐Item Short Form Health Survey, Ware Jr. et al., 1992) | -0.4 |  |
| Lee et al. (2020) | 180 | 100 | 39.7 | 6.38 | No data | Cross-sectional | East Asia | Parental / familial (scale developed by Lee & Lee, 1997) | Health-related quality of life (12‐Item Short Form Health Survey, Ware Jr. et al., 1996) | -0.57 |  |
| Limbers et al. (2020) | 200 | 100 | 33.5 | No data | No data | Cross-sectional | North America | Parental / familial (Parenting Stress Scale, Berry & Jones, 1995) | Quality of life (World Health Organization Quality of Life Assessment-BREF, WHO, 1996) | -0.28 |  |
| Loh et al. (2017) | 194 | 100 | 35 | No data | No data | Cross-sectional | Australia and New Zeeland | Parental / familial (Parenting Stress Scale, Berry & Jones, 1995) | Satisfaction with life (The Quality of Life Enjoyment and Satisfaction Questionanaire, Endicott et al., 1993) | -0.44 |  |
| Lu et al. (2018) | 479 | 63.1 | 36.59 | 6.68 | No data | Cross-sectional | East Asia | Parental / familial (Parent Stress Index Short Form, Abidin, 1995) | Satisfaction with life (Satisfaction with Life Scale, Diener et al., 1985) | -0.391 |  |
| Lubiewska & Derbis (2016) | 575 | 100 | 43.06 | No data | No data | Cross-sectional | Europe | Parental / familial (The Child Rearing Stress Scale, Gerris et al., 1998) | Satisfaction with life (Satisfaction with Life Scale, Diener et al., 1985) | -0.22 |  |
| Matalon et al. (2022) | 422 | 50 | 37.4 | 4.56 | 46.9 | Cross-sectional | Middle East | Parental / familial (Parent Stress Index Short Form, Abidin, 1995) | Satisfaction with life (Satisfaction with Life Scale, Diener et al., 1985) | -0.525 |  |
| Mazumdar et al. (2021) | 242 | 100 | 35.5 | No data | No data | Cross-sectional | South Asia | Parental / familial (Parenting Stress Scale, Berry & Jones, 1995) | Well-being (Psychological Well-Being Scale–Short, Ryff, 1989) | -0.544 |  |
| Miski Aydin et al. (2023) | 258 | 100 | 36.2 | No data | No data | Cross-sectional | Europe | General (Perceived Stress Scale, Cohen et al., 1983) | Well-being (Employee well-being questionnaire, Zheng et al., 2015) | -0.609 |  |
| Moreira et al. (2013) | 104 | 90.4 | 41.98 | 12.44 | 55.8 | Cross-sectional | Europe | Parental / familial (Parent Stress Index Short Form, Abidin, 1995) | Quality of life (EUROHIS-QOL 8-item index,  Schmidt et al. 2006) | -0.49 |  |
|  | 142 | 95.8 | 41.54 | 11.79 | 54.9 | Cross-sectional | Europe | Parental / familial (Parent Stress Index Short Form, Abidin, 1995) | Quality of life (EUROHIS-QOL 8-item index,  Schmidt et al. 2006) | -0.52 |  |
| Nachshen & Minnes (2005) | 100 | 97 | 40.32 | 8.61 | 30 | Cross-sectional | North America | Parental / familial (Parenting Stress Index, Abidin, 1986) | Emotional well-being (The Family Member Well-Being Index, McCubbin & Patterson 1982) | -0.59 |  |
|  | 100 | 98 | 37.67 | 8.31 | 50 | Cross-sectional | North America | Parental / familial (Parenting Stress Index, Abidin, 1986) | Emotional well-being (The Family Member Well-Being Index, McCubbin & Patterson 1982) | -0.55 |  |
| Neff & Faso (2015) | 51 | 78.43 | 40.41 | No data | No data | Cross-sectional | North America | Parental / familial (Parent Stress Index Short Form, Abidin, 1995) | Satisfaction with life (Satisfaction with Life Scale, Diener et al., 1985) | -0.52 |  |
| Nelson-Coffey et al. (2019) | 140 | No data | No data | No data | No data | Cross-sectional | North America | General (Hassles and Uplifts Scale DeLongis et al., 1988) | Satisfaction with life (Satisfaction with Life Scale, Diener et al., 1985) | -0.276 |  |
| Nordheim et al. (2018) | 59 | 52.5 | No data | No data | No data | Cross-sectional | Europe | Parental / familial (Parent Stress Index Short Form, Abidin, 1995) | Quality of life (traslated The Quality of Life, Burckhardt & Anderson, 2003) |  | -0.018 |
| Papadopoulos et al. (2023) | 53 | 100 | 39.8 | 4.49 | 20.75 | Cross-sectional | Europe | Parental / familial (Autism Parenting Stress Index, Silva & Schalock, 2012) | Quality of life (Beach Center Family Quality of Life Scale, Park et al., 2003) | -0.435 |  |
| Rai & Rani (2019) | 135 | 100 | No data | 0.5 | No data | Cross-sectional | South Asia | Parental / familial (Parental Stressor Scale: Neonatal Intensive Care Unit, Milles et al., 1993) | Health-related quality of life (36‐Item Short Form Health Survey, Ware Jr. et al., 1992) | -0.467 |  |
| Rambod et al. (2023) | 130 | 67.7 | No data | No data | No data | Cross-sectional | Middle East | Parental / familial (Parental Stressor Scale: Neonatal Intensive Care Unit, Milles et al., 1993) | Well-being (Psychological Well-Being Scale–Short, Ryff, 1989) | -0.31 |  |
| Rohde et al. (2022) | 561 | 78 | 45 | No data | No data | Cross-sectional | Europe | General (Perceived Stress Questionnaire, Levenstein et al., 1993) | Health-related quality of life (36‐Item Short Form Health Survey, Ware Jr. et al., 1992) | -0.73 |  |
| Salami (2007) | 480 | 100 | 35.4 | No data | No data | Cross-sectional | Africa | Other (multiple role strain, scale developed by Hassan, 2004) | Well-being (Psychological Well-Being Scale–Short, Ryff, 1989) | -0.35 |  |
| Samadi et al. (2013) | 37 | 64.9 | No data | No data | 29.72 | Intervention | Middle East | Parental / familial (Parent Stress Index Short Form, Abidin, 1995) | Health-related quality of life (General Health Questionnaire, Goldberg and Williams, 1988) | 0.676 |  |
| Savari et al. (2021) | 250 | 72.8 | No data | No data | No data | Cross-sectional | Middle East | General (Perceived Stress Scale, Cohen et al., 1983) | Quality of life (World Health Organization Quality of Life Assessment-BREF, WHO, 1996) | -0.48 |  |
| Sharda (2023) | 139 | 90.6 | 42.73 | No data | No data | Cross-sectional | North America | Parental / familial (Parenting Stress Scale, Berry & Jones, 1995) | Well-being (Mental Health Continuum Short Form, Keyes, 2009) | -0.477 |  |
| Sharkia & Taubman-Ben-Ari (2024) | 77 | 100 | 28.53 | 1 | No data | Cross-sectional | Middle East | General (Perceived Stress Scale, Cohen et al., 1983) | Satisfaction with life (Satisfaction with Life Scale, Diener et al., 1985) | -0.38 |  |
|  | 77 | 100 | 27.85 | 1 | No data | Cross-sectional | Middle East | General (Perceived Stress Scale, Cohen et al., 1983) | Satisfaction with life (Satisfaction with Life Scale, Diener et al., 1985) | -0.44 |  |
| Silva et al. (2018) | 238 | 81.9 | 44.89 | No data | No data | Cross-sectional | Europe | Parental / familial (e Effects on Parents scale of the QoLISSY questionnaire, The European QoLISSY Group, 2013 | Quality of life (EUROHIS-QOL 8-item index,  Schmidt et al. 2006) | -0.34 |  |
| Skok et al. (2006) | 43 | 100 | No data | 8.82 | 42 | Cross-sectional | Australia and New Zeeland | General (Perceived Stress Scale, Cohen et al., 1983) | Satisfaction with life (Satisfaction with Life Scale, Diener et al., 1985) | -0.59 |  |
| Staunton et al. (2020) | 33 | No data | No data | 13 | 27 | Cross-sectional | Europe | Parental / familial (Parenting Stress Index, Abidin, 1986) | Quality of life (Family Quality of Life Scale, Hoffman et al. 2006) | -0.405 |  |
| Streisand et al. (2010) | 278 | 100 | No data | 12.1 | 46 | Cross-sectional | North America | Parental / familial (self-created) | Well-being (self-created) | 0.19 |  |
| Sun et al. (2024) | 231 | 100 | 39.7 | No data | No data | Longitudinal | North America | Parental / familial (Parenting Events Scale, Crnic & Booth, 1991) | Satisfaction with life (Satisfaction with Life Scale, Diener et al., 1985) | -0.11 |  |
| Tan et al. (2023) | 214 | 51.9 | 46.39 | No data | No data | Cross-sectional | East Asia | Parental / familial (Parenting Stress Scale, Berry & Jones, 1995) | Well-being (World Health Organization Well-Being Index, WHO, 1998) | -0.383 |  |
| Thorsteinsen et al. (2024) | 448 | 51.33 | 41.18 | No data | No data | Cross-sectional | Europe | Other (Covid-related, developed by Kvalø et al., 2024) | Well-being (World Health Organization Well-Being Index, WHO, 1998) | -0.223 |  |
| Tien et al. (2022) | 128 | 0 | No data | No data | No data | Cross-sectional | East Asia | Parental / familial (Parenting Stress Scale, Berry & Jones, 1995) | Quality of life (World Health Organization Quality of Life Assessment-BREF, WHO, 1996) | -0.35 |  |
| Vahedparast et al. (2022) | 192 | 50 | 51 | 10.4 | 28 | Cross-sectional | Middle East | Parental / familial (Parent Stress Index Short Form, Abidin, 1995) | Quality of life (Mental quality of life scale from 36‐Item Short Form Health Survey, Ware Jr. et al., 1992) | -0.361 |  |
| Wang et al. (2017) | 369 | 100 | 33.59 | No data | No data | Cross-sectional | East Asia | Parental / familial (Parent Stress Index Short Form, Abidin, 1995) | Satisfaction with life (Satisfaction with Life Scale, Diener et al., 1985) | -0.405 |  |
| Wang et al. (2020) | 420 | 50 | 39.97 | 10.09 | 16.66 | Cross-sectional | East Asia | Parental / familial (Parent Stress Index Short Form, Abidin, 1995) | Emotional Quality of life (dimension form the Beach Center Family Quality of Life Scale, Park et al., 2003) | -0.315 |  |
| Witt et al. (2011) | 587 | 100 | 37.17 | 5.1 | 54 | Cross-sectional | North America | General (Calgary Symptoms of Stress Inventory, Carlson & Thomas, 2007) | Health-related quality of life (12‐Item Short Form Health Survey, Ware Jr. et al., 1996) |  | -0.2 |
| You et al. (2018) | 242 | 100 | 33.97 | 10.75 | 70.2 | Cross-sectional | East Asia | Parental / familial (Parent Stress Index Short Form, Abidin, 1995) | Satisfaction with life (Satisfaction with Life Scale, Diener et al., 1985) | -0.5 |  |
| Zeng et al. (2021) | 438 | 50 | 40.65 | 10.3 | 16.5 | Cross-sectional | East Asia | Parental / familial (Parent Stress Index Short Form, Abidin, 1995) | Emotional Quality of life (dimension form the Beach Center Family Quality of Life Scale, Park et al., 2003) | -0.388 |  |
| Zhang et al. (2022) | 494 | No data | No data | 5.08 | 41.7 | Cross-sectional | East Asia | Parental / familial (Parenting Daily Hassles Scale, Crnic & Greenberg, 1990) | Emotional well-being (Scale of Positive and Negative Experiences, Diener et al., 2010) | -0.2 |  |

References (All studies that were included in the final analysis are indicated with an ^*^ in our list of references).

Abidin, R. R. (1986). *Parenting Stress Index. Odessa, FL, USA:* Psychological Assessment Resources.

Abidin, R. R. (1995). *Parenting stress index* (3rd ed.). Odessa, FL: Psychological Assessment Resources.

*Alhuzimi, T. (2021). Stress and emotional wellbeing of parents due to change in routine for children with Autism Spectrum Disorder (ASD) at home during COVID-19 pandemic in Saudi Arabia. *Research in Developmental Disabilities*, *108*, 103822. https://doi.org/10.1016/j.ridd.2020.103822

*Angeline, J., & Rathnasabapathy, M. (2023). Role of Perceived Social Support in the Relationship between Parenting Stress and Psychological Well-Being of Mothers of Children with ADHD: A Mediation Model. *Universal Journal of Public Health*, *11*(6), 838–844. https://doi.org/10.13189/ujph.2023.110607

*Augustijn, L. (2023). Joint Physical Custody and Mothers’ Well-Being. An Analysis of Life Satisfaction, Depressiveness, and Stress. *Applied Research in Quality of Life*, *18*(5), 2371–2395. https://doi.org/10.1007/s11482-023-10190-z

*Bae, S. Y., Chang, P.-J., & Lee, C.-K. (2020). Structural Relationships among Online Community Use, Parental Stress, Social Support, and Quality of Life between Korean and Taiwanese Employed Mothers. *Sustainability*, *12*(24), 10681. https://doi.org/10.3390/su122410681

*Bailey, D. B., Sideris, J., Roberts, J., & Hatton, D. (2008). Child and genetic variables associated with maternal adaptation to fragile X syndrome: A multidimensional analysis. *American Journal of Medical Genetics Part A*, *146A*(6), 720–729. https://doi.org/10.1002/ajmg.a.32240

*Bang, K.-S., & Jang, S.-Y. (2022). Influence of parenting role sharing, parenting stress, and happiness on warm parenting behavior in mothers of children aged 6 years: An analysis using data from the seventh panel study on Korean children. *Child Health Nursing Research*, *28*(1), 82–90. https://doi.org/10.4094/chnr.2022.28.1.82

*Barnett, R. C., & Gareis, K. C. (2006). Parental After-School Stress and Psychological Well-Being. *Journal of Marriage and Family*, *68*(1), 101–108. https://doi.org/10.1111/j.1741-3737.2006.00236.x

Berry, J. O., & Jones, W. H. (1995). The Parental Stress Scale: Initial Psychometric Evidence. *Journal of Social and Personal Relationships*, *12*(3), 463–472. https://doi.org/10.1177/0265407595123009

Blumberg, S. J., Foster, E. B., Frasier, A. M., Satorius, J., Skalland, B. J., Nysse-Carris, K. L., Morrison, H. M., Chowdhury, S. R., & O’Connor, K. S. (2012). Design and operation of the National Survey of Children’s Health, 2007. *Vital and Health Statistics. Ser. 1, Programs and Collection Procedures*, *55*, 1–149.

*Bohadana, G., Morrissey, S., & Paynter, J. (2019). Self-compassion: A Novel Predictor of Stress and Quality of Life in Parents of Children with Autism Spectrum Disorder. *Journal of Autism and Developmental Disorders*, *49*(10), 4039–4052. https://doi.org/10.1007/s10803-019-04121-x

*Bowen, A., Chen, Y. M., Kodam, R. S., Odoi, J. A., & Anto-Ocrah, M. (2022). “At Least Somebody Sees You as a Hero”: Fatherhood Stress and Well-Being in Ghana. *American Journal of Men’s Health*, *16*(6), 155798832211381. https://doi.org/10.1177/15579883221138185

*Brelsford, G. M., & Doheny, K. K. (2022). Parents’ Spiritual Struggles and Stress: Associations With Mental Health and Cognitive Well-Being Following a Neonatal Intensive Care Unit Experience. *Psychology of Religion and Spirituality*, *14*(1), 119–127. https://doi.org/10.1037/rel0000381

Brouwer, W. B. F., Van Exel, N. J. A., Van Gorp, B., & Redekop, W. K. (2006). The CarerQOL instrument: a new instrument to measure care related quality of life of informal caregivers for use in economic evaluations. Quality of Life Research, 15(6), 1005-1021.

Burckhardt, C. S., & Anderson, K. L. (2003). The Quality of Life Scale (QOLS): Reliability, Validity, and Utilization. *Health and Quality of Life Outcomes*, *1*(1), 60. https://doi.org/10.1186/1477-7525-1-60

Butler, J., & Kern, M. L. (2016). The PERMA-Profiler: A brief multidimensional measure of flourishing. *International Journal of Wellbeing*, *6*(3), 1–48. https://doi.org/10.5502/ijw.v6i3.526

*Calderwood, C., Breaux, R., Ten Brummelhuis, L. L., Mitropoulos, T., & Swanson, C. S. (2022). When daily challenges become too much during COVID-19: Implications of family and work demands for work–life balance among parents of children with special needs. *Journal of Occupational Health Psychology*, *27*(5), 516–527. https://doi.org/10.1037/ocp0000333

Cardeña, E., Koopman, C., Classen, C., Waelde, L. C., & Spiegel, D. (2000). Psychometric Properties of the Stanford Acute Stress Reaction Questionnaire (SASRQ): A Valid and Reliable Measure of Acute Stress. *Journal of Traumatic Stress*, *13*(4), 719–734. https://doi.org/10.1023/A:1007822603186

Carlson, L. E., & Thomas, B. C. (2007). Development of the calgary symptoms of stress inventory (c-sosi). *International Journal of Behavioral Medicine*, *14*(4), 249–256. https://doi.org/10.1007/BF03003000

*Casino-García, A. M., Llinares-Insa, L. I., Guillén-Martín, V. M., & Ibáñez-García, A. (2024). Giftedness and Family Well-being: The Role of Emotional Intelligence, Perceived Social Support and Stress. *Psicothema*, *36*(3), 277–286. https://doi.org/10.7334/psicothema2023.401

Cella, D., Riley, W., Stone, A., Rothrock, N., Reeve, B., Yount, S., Amtmann, D., Bode, R., Buysse, D., Choi, S., Cook, K., DeVellis, R., DeWalt, D., Fries, J. F., Gershon, R., Hahn, E. A., Lai, J.-S., Pilkonis, P., Revicki, D., … Hays, R. (2010). The Patient-Reported Outcomes Measurement Information System (PROMIS) developed and tested its first wave of adult self-reported health outcome item banks: 2005–2008. *Journal of Clinical Epidemiology*, *63*(11), 1179–1194. https://doi.org/10.1016/j.jclinepi.2010.04.011

*Cernvall, M., Ljungman, L., Wikman, A., Carlbring, P., Ljungman, G., & von Essen, L. (2016). Dimensions of Posttraumatic Stress Symptoms and their Relationships with Depression, Anxiety, and Quality of Life in Parents of Children Recently Diagnosed with Cancer. *Psycho-Oncology*, *25*(SP. S3), 76–76.

*Cham, C. Q., Ibrahim, N., Kalaman, C. R., Ho, M. C., Visvalingam, U., Ahmad Shahabuddin, F., Abd Rahman, F. N., Halim, M. R. T. A., Harbajan Singh, M. K., Azhar, F. L., Yahya, A. N., Roslan, S., & Siau, C. S. (2024). Factors associated with depression, anxiety, and satisfaction with life among Malaysian parental caregivers of adolescent psychiatric patients: A cross-sectional study. *Cambridge Prisms: Global Mental Health*, *11*, e12. https://doi.org/10.1017/gmh.2024.5

*Chan, R. C. H. (2022). Dyadic associations between COVID‐19‐related stress and mental well‐being among parents and children in Hong Kong: An actor–partner interdependence model approach. *Family Process*, *61*(4), 1730–1748. https://doi.org/10.1111/famp.12760

*Cheung, R. Y. M., Leung, S. S. W., & Mak, W. W. S. (2019). Role of Mindful Parenting, Affiliate Stigma, and Parents’ Well-being in the Behavioral Adjustment of Children with Autism Spectrum Disorder: Testing Parenting Stress as a Mediator. *Mindfulness*, *10*(11), 2352–2362. https://doi.org/10.1007/s12671-019-01208-5

*Chu, S. Y., Park, H., Lee, J., Shaharuddin, K. K. binti, & Gan, C. H. (2020). Self-stigma and its associations with stress and quality of life among Malaysian parents of children with autism. *Child Care Health and Development*, *46*(4), 485–494. https://doi.org/10.1111/cch.12771

Cohen, A. K., Hoyt, L. T., & Dull, B. (2020). A Descriptive Study of COVID-19–Related Experiences and Perspectives of a National Sample of College Students in Spring 2020. *Journal of Adolescent Health*, *67*(3), 369–375. https://doi.org/10.1016/j.jadohealth.2020.06.009

Cohen, S., Kamarck, T., & Mermelstein, R. (1983). A Global Measure of Perceived Stress. *Journal of Health and Social Behavior*, *24*(4), 385. https://doi.org/10.2307/2136404

Crnic, K. A., & Booth, C. L. (1991). Mothers’ and Fathers’ Perceptions of Daily Hassles of Parenting across Early Childhood. *Journal of Marriage and Family*, *53*(4), 1042–1050. https://doi.org/10.2307/353007

Crnic, K. A., & Greenberg, M. T. (1990). Minor Parenting Stresses with Young Children. *Child Development*, *61*(5), 1628–1637. https://doi.org/10.1111/j.1467-8624.1990.tb02889.x

*Dardas, L. A., & Ahmad, M. M. (2015a). Coping Strategies as Mediators and Moderators between Stress and Quality of Life among Parents of Children with Autistic Disorder. *Stress and Health*, *31*(1), 5–12. https://doi.org/10.1002/smi.2513

*Dardas, L. A., & Ahmad, M. M. (2015b). For fathers raising children with autism, do coping strategies mediate or moderate the relationship between parenting stress and quality of life? *Research in Developmental Disabilities*, *36*, 620–629. https://doi.org/10.1016/j.ridd.2014.10.047

*Dellve, L., Samuelsson, L., Tallborn, A., Fasth, A., & Hallberg, L. R. M. (2006). Stress and well-being among parents of children with rare diseases: A prospective intervention study. *Journal of Advanced Nursing*, *53*(4), 392–402. https://doi.org/10.1111/j.1365-2648.2006.03736.x

DeLongis, A., Folkman, S., & Lazarus, R. S. (1988). The impact of daily stress on health and mood: Psychological and social resources as mediators. *Journal of Personality and Social Psychology*, *54*(3), 486–495. https://doi.org/10.1037/0022-3514.54.3.486

Derogatis, L. R. (1993). Brief symptom inventory. *European Journal of Psychological Assessment*.

Diener, E., Emmons, R. A., Larsen, R. J., & Griffin S. (1985). The Satisfaction with Life Scale. Journal of Personality Assessment, 49(1), 71-75.

Diener, E., Wirtz, D., Tov, W., Kim-Prieto, C., Choi, D. W., Oishi, S., & Biswas-Diener, R. (2010). New well-being measures: Short scales to assess flourishing and positive and negative feelings. Social Indicators Research, 97, 143-156. https://doi.org/10.1007/s11205-009-9493-y

*Dijkstra-de Neijs, L., Boeke, D. B., Van Berckelaer-Onnes, I. A., Swaab, H., & Ester, W. A. (2024). Parental Stress and Quality of Life in Parents of Young Children with Autism. *Child Psychiatry & Human Development*. https://doi.org/10.1007/s10578-024-01693-3

*Droogmans, G., Vergaelen, E., Van Buggenhout, G., & Swillen, A. (2021). Stressed parents, happy parents. An assessment of parenting stress and family quality of life in families with a child with Phelan‐McDermid syndrome. *Journal of Applied Research in Intellectual Disabilities*, *34*(4), 1076–1088. https://doi.org/10.1111/jar.12858

Eapen, V., Črnčec, R., Walter, A., & Tay, K. P. (2014). Conceptualisation and development of a quality of life measure for parents of children with autism spectrum disorder. Autism Research and Treatment, 2014, 1–11. https://doi.org/10.1155/2014/924182.

Ehrle, J., & Moore, K. A. (1997). *1997 NSAF Benchmarking Measures of Child and Family Well-Being*. Urban Institute.

*Ekas, N. V., & Whitman, T. L. (2011). Adaptation to Daily Stress Among Mothers of Children With an Autism Spectrum Disorder: The Role of Daily Positive Affect. *Journal of Autism and Developmental Disorders*, *41*(9), 1202–1213. https://doi.org/10.1007/s10803-010-1142-4

*Emam, M. M., Al-Hendawi, M., & Gaafar Ali, D. (2021). Parenting stress and life satisfaction in families of children with disabilities: The mediating effect of social support in three Arab speaking countries. *Journal of Family Studies*, 1–19. https://doi.org/10.1080/13229400.2021.1893791

Endicott, J., Nee, J., Harrison, W., & Blumenthal, R. (1993). Quality of Life Enjoyment and Satisfaction Questionnaire: a new measure. *Psychopharmacology Bulletin*, *29*(2), 321–326.

*Eugene, D. R., Blalock, C., Robinson, E. D., & Crutchfield, J. (2022). The moderating effect of COVID-19 stress on school racial climate and parent and child mental well-being. *Children and Youth Services Review*, *139*, 106572. https://doi.org/10.1016/j.childyouth.2022.106572

*Everhart, R. S., Fiese, B. H., & Smyth, J. M. (2008). A Cumulative Risk Model Predicting Caregiver Quality of Life in Pediatric Asthma. *Journal of Pediatric Psychology*, *33*(8), 809–818. https://doi.org/10.1093/jpepsy/jsn028

*Faden, S. Y., Merdad, N., & Faden, Y. A. (2023). Parents of Children With Neurodevelopmental Disorders: A Mixed Methods Approach to Understanding Quality of Life, Stress, and Perceived Social Support. *Cureus*. https://doi.org/10.7759/cureus.37356

Fatima, M. (2018). *Emotional Intelligence, Caregiver stress and Social Support as Predictors of Marital Adjustment in the Mothers of Children with Physical Disability* [Doctoral dissertation]. University of Sargodha.

*Fidika, A., Salewski, C., & Goldbeck, L. (2013). Quality of life among parents of children with phenylketonuria (PKU). *Health and Quality of Life Outcomes*, *11*(1), 54. https://doi.org/10.1186/1477-7525-11-54

*Findler, L., Jacoby, A. K., & Gabis, L. (2016). Subjective happiness among mothers of children with disabilities: The role of stress, attachment, guilt and social support. *Research in Developmental Disabilities*, *55*, 44–54. https://doi.org/10.1016/j.ridd.2016.03.006

Frisch MB. (1994). Quality of life inventory. Minneapolis: National Computer Systems, Inc.

*Gavín-Chocano, Ó., García-Martínez, I., Torres-Luque, V., & Checa-Domene, L. (2024). Resilient Moderating Effect between Stress and Life Satisfaction of Mothers and Fathers with Children with Developmental Disorders Who Present Temporary or Permanent Needs. *European Journal of Investigation in Health, Psychology and Education*, *14*(3), 474–487. https://doi.org/10.3390/ejihpe14030032

Gerris, J. R. M., Houtmans, M. J. M., Kwaaitaal-Roosen, E. M. G., de Schipper, J. C., Vermulst, A. A., Janssens, J. M. A. M. (1998). Parents, adolescents and young adults in Dutch families: A longitudinal study. Nijmegen: Institute of Family Studies.

*Gerstein, E. D., Crnic, K. A., Blacher, J., & Baker, B. L. (2009). Resilience and the course of daily parenting stress in families of young children with intellectual disabilities. *Journal of Intellectual Disability Research*, *53*(12), 981–997. https://doi.org/10.1111/j.1365-2788.2009.01220.x

Goldberg, D. P., and Williams, P. (1988). *A Users’ Guide To The General Health Questionnaire.* London: GL Assessment

*Gómez-Ortiz, O., Rubio, A., Roldán-Barrios, A., Ridao, P., & López-Verdugo, e I. (2023). Parental stress and life satisfaction: A comparative study of social services users and nonusers from a gender perspective. *Journal of Community Psychology*, *51*(1), 345–360. https://doi.org/10.1002/jcop.22907

Grossi, E., Groth, N., Mosconi, P., Cerutti, R., Pace, F., Compare, A., & Apolone, G. (2006). Development and validation of the short version of the Psychological General Well-Being Index (PGWB-S). *Health and Quality of Life Outcomes*, *4*(1), 88. https://doi.org/10.1186/1477-7525-4-88

*Hakimzadeh, A., Mousavizadeh, S. N., Shafighi, A., & Tehrani, F. J. (2023). Evaluation of Parental Stress and Quality of Life Among Iranian Parents of Hearing-Impaired Children with Cochlear Implantation History: A Cross-Sectional and Descriptive-Correlational Study. *Journal of Comprehensive Pediatrics*, *15*(1).

*Han, J.-W., Yang, B., & Lee, H. (2024). Serial Dual Mediating Effects of Parenting Stress on Life Satisfaction among Parents of School-Aged Children with Chronic Conditions. *Healthcare*, *12*(4), 461. https://doi.org/10.3390/healthcare12040461

Hassan, E. (2004). *Psychosocial correlates of women occupational commitment and work - family conflict in selected organizations in Ogun and Lagos States of Nigeria*.

*Henton, S., & Swanson, V. (2023). A mixed-methods analysis of the role of online social support to promote psychological wellbeing in new mothers. *DIGITAL HEALTH*, *9*, 205520762211474. https://doi.org/10.1177/20552076221147433

Hoffman, L., Marquis, J., Poston, D., Summers, J. A., & Turnbull, A. (2006). Assessing Family Outcomes: Psychometric Evaluation of the Beach Center Family Quality of Life Scale. *Journal of Marriage and Family*, *68*(4), 1069–1083. https://doi.org/10.1111/j.1741-3737.2006.00314.x

*Hsiao, Y.-J. (2016). Pathways to mental health-related quality of life for parents of children with autism spectrum disorder: Roles of parental stress, children’s performance, medical support, and neighbor support. *Research in Autism Spectrum Disorders*, *23*, 122–130. https://doi.org/10.1016/j.rasd.2015.10.008

*Hsiao, Y.-J. (2024). Parental stress, family resilience, and health-related quality of life: parents of children with autism spectrum disorder. *Current Psychology*. https://doi.org/10.1007/s12144-024-06687-x

*Huang, Y.-P., Chang, M., Chi, Y.-L., & Lai, F.-C. (2013). Health-related quality of life in fathers of children with or without developmental disability: The mediating effect of parental stress. *Quality of Life Research*, *23*(1), 175–183. https://doi.org/10.1007/s11136-013-0469-7

Ibrahim, N., Wong, A., Cham, C. Q., Chu, S. Y., Kalaman, C. R., & Siau, C. S. (2023). Translation and validation of the Malay perceived stress scale modified for covid-19. *The Malaysian Journal of Medical Sciences: MJMS*, *30*(2), 161. https://doi.org/10.21315/mjms2023.30.2.15

*Irannejad, F., Dehghan, M., & Rabori, R. M. (2018). Stress and quality of life in parents of children with phenylketonuria. *Journal of Child and Adolescent Psychiatric Nursing*, *31*(2–3), 48–52. https://doi.org/10.1111/jcap.12207

*Javed, H., & Zahid, S. (2021). Impact of social support on caregiver stress and life satisfaction in mothers of children with neurodevelopmental disorders. *Rawal Medical Journal*, *46*(3).

*Johnson, N., Frenn, M., Feetham, S., & Simpson, P. (2011). Autism Spectrum Disorder: Parenting Stress, Family Functioning and Health-Related Quality of Life. *Families Systems & Health*, *29*(3), 232–252. https://doi.org/10.1037/a0025341

Juniper, E. F., Guyatt, G. H., Feeny, D. H., Ferrie, P. J., Griffith, L. E., & Townsend, M. (1996). Measuring quality of life in the parents of children with asthma. *Quality of Life Research*, *5*(1), 27–34. https://doi.org/10.1007/BF00435966

Kammann, R., & Flett, R. (1983). Sourcebook for measuring well-being with Affectometer 2. Dunedin, New Zealand: Why Not? Foundation.

*Kamran, F., Tajalli, S., Ebadi, A., Sagheb, S., Fallahi, M., & Kenner, C. (2023). Quality of life and stress in mothers of preterm infant with feeding problems: A cross sectional study. *Journal of Neonatal Nursing*, *29*(1), 68–74. https://doi.org/10.1016/j.jnn.2022.02.006

Kanner, A. D., Coyne, J. C., Schaefer, C., & Lazarus, R. S. (1981). Comparison of two modes of stress measurement: Daily hassles and uplifts versus major life events. *Journal of Behavioral Medicine*, *4*(1), 1–39. https://doi.org/10.1007/BF00844845

*Kaugars, A., Shields, C., & Brosig, C. (2018). Stress and quality of life among parents of children with congenital heart disease referred for psychological services. *Congenital Heart Disease*, *13*(1), 72–78. https://doi.org/10.1111/chd.12547

Keyes, C. L. M. (2002). The mental health continuum: from languishing to flourishing in life. Journal of Health and Social Behavior, 43(2),207–222. https://doi.org/10.2307/3090197.

Keyes, C. L. M. (2009). Brief description of the mental health continuum short form (MHC-SF). Retrieved 18 March, 2019, from http://www.sociology.emory.edu/ckeyes/

*Khusaifan, S. J., & El Keshky, M. E. S. (2021). Social Support as a Protective Factor for the Well-Being of Parents of Children with Autism in Saudi Arabia. *Journal of Pediatric Nursing*, *58*, e1–e7. https://doi.org/10.1016/j.pedn.2020.11.014

Kim, K.-H., & Kang. (1997). Development of the parenting stress scale. *Journal of the Korean Home Economics Association*, *35*(5), 141.

*Kim, Y.-J., & Mitrani, V. B. (2019). Impact of household composition and family functioning on health of Hispanic mothers in mental health treatment: A secondary analysis study. *Journal of Psychiatric and Mental Health Nursing*, *26*(1–2), 11–18. https://doi.org/10.1111/jpm.12500

*King, R. M., Knibb, R. C., & Hourihane, J. O. (2009). Impact of peanut allergy on quality of life, stress and anxiety in the family. *Allergy*, *64*(3), 461–468. https://doi.org/10.1111/j.1398-9995.2008.01843.x

Kvalø, M., Parks-Stamm, E. J., Thorsteinsen, K., Olsen, M., & Martiny, S. E. (2024). Mapping the well-being of Norwegian mothers during the COVID-19 pandemic [Manuscript submitted for publication].

*Kumar, K., Sharma, R., Saini, L., Shah, R., Sharma, A., & Mehra, A. (2022). Personification of stress, depression, anxiety, and quality of life, among parents of attention deficit hyperkinetic disorder children. *Indian Journal of Social Psychiatry*, *38*(2), 137. https://doi.org/10.4103/ijsp.ijsp_116_20

*Lamis, D. A., Wilson, C. K., Tarantino, N., Lansford, J. E., & Kaslow, N. J. (2014). Neighborhood disorder, spiritual well-being, and parenting stress in African American women. *Journal of Family Psychology*, *28*(6), 769–778. https://doi.org/10.1037/a0036373

Lee, J. W. & Lee, H. J. (1997). A study on the stress and coping patterns of mothers with cerebral palsy children. Korean Journal of Child Health Nursing, 3(2), 190‐202.

*Lee, M. H., Matthews, A. K., Park, C. G., Vincent, C., Hsieh, K., & Savage, T. A. (2020). Relationships among parenting stress, health-promoting behaviors, and health-related quality of life in Korean mothers of children with cerebral palsy. *Research in Nursing & Health*, *43*(6), 590–601. https://doi.org/10.1002/nur.22074

*Lee, S.-Y., & Hsu, H.-C. (2012). Stress and health-related well-being among mothers with a low birth weight infant: The role of sleep. *Social Science & Medicine*, *74*(7), 958–965. https://doi.org/10.1016/j.socscimed.2011.12.030

Levenstein, S., Prantera, C., Varvo, V., Scribano, M. L., Berto, E., Luzi, C., & Andreoli, A. (1993). Development of the perceived stress questionnaire: A new tool for psychosomatic research. *Journal of Psychosomatic Research*, *37*(1), 19–32. https://doi.org/10.1016/0022-3999(93)90120-5

*Limbers, C. A., McCollum, C., & Greenwood, E. (2020). Physical activity moderates the association between parenting stress and quality of life in working mothers during the COVID-19 pandemic. *Mental Health and Physical Activity*, *19*, 100358. https://doi.org/10.1016/j.mhpa.2020.100358

*Loh, J., Harms, C., & Harman, B. (2017). Effects of Parental Stress, Optimism, and Health-Promoting Behaviors on the Quality of Life of Primiparous and Multiparous Mothers. *Nursing Research*, *66*(3), 231–239. https://doi.org/10.1097/NNR.0000000000000219

Lovibond, S. H., & Lovibond, P. F. (1995). *Depression Anxiety Stress Scales* (2nd ed.). Psychology Foundation

*Lu, M.-H., Wang, G.-H., Lei, H., Shi, M.-L., Zhu, R., & Jiang, F. (2018). Social Support as Mediator and Moderator of the Relationship Between Parenting Stress and Life Satisfaction Among the Chinese Parents of Children with ASD. *Journal of Autism and Developmental Disorders*, *48*(4), 1181–1188. https://doi.org/10.1007/s10803-017-3448-y

*Lubiewska, K., & Derbis, R. (2016). Relations between Parenting Stress, Attachment, and Life Satisfaction in Mothers of Adolescent Children. *Polish Journal of Applied Psychology*, *14*(2), 87–112. https://doi.org/10.1515/pjap-2015-0056

Lyubomirsky, S., & Lepper, H. S. (1999). A Measure of Subjective Happiness: Preliminary Reliability and Construct Validation. *Social Indicators Research*, *46*(2), 137–155. https://doi.org/10.1023/A:1006824100041

*Matalon, C., Turliuc, M. N., & Mairean, C. (2022). Children’s opposition, marital and life satisfaction: The mediating role of parenting stress. *Family Relations*, *71*(4), 1785–1801. https://doi.org/10.1111/fare.12670

*Mazumdar, K., Sen, I., Gupta, P., & Parekh, S. (2021). Psychological Well-Being of Indian Mothers During the COVID-19 Pandemic: The Roles of Self-Compassion, Psychological Inflexibility, and Parenting Stress. *International Perspectives in Psychology*, *10*(3), 155–162. https://doi.org/10.1027/2157-3891/a000024

McCubbin, H., & Patterson, J. (1982). Family member wellbeing index. *Family Assessment: Resiliency, Coping and Adaptation: Inventories for Research and Practice*, 774–783.

Milles, M.S., Funk, S.G., Carlson, J., (1993). May-Jun. Parental Stressor Scale: neonatal intensive care unit. Nurs. Res. 42 (3), 148–152.

*Miski Aydin, E., Metin-Orta, I., Metin-Camgoz, S., & Aksan, N. (2023). Does Overparenting Hurt Working Turkish Mother’s Well-being? The Influence of Family–Work Conflict and Perceived Stress in Established Adulthood. *Journal of Adult Development*, *30*(1), 131–144. https://doi.org/10.1007/s10804-022-09408-5

*Moreira, H., Frontini, R., Bullinger, M., & Canavarro, M. C. (2013). Caring for a child with Type 1 diabetes: Links between family cohesion, perceived impact, and parental adjustment. *Journal of Family Psychology*, *27*(5), 731–742. https://doi.org/10.1037/a0034198

Motowidlo, S. J., Packard, J. S., & Manning, M. R. (1986). Occupational stress: Its causes and consequences for job performance. *Journal of Applied Psychology*, *71*(4), 618–629. https://doi.org/10.1037/0021-9010.71.4.618

*Nachshen, J. S., & Minnes, P. (2005). Empowerment in parents of school-aged children with and without developmental disabilities. *Journal of Intellectual Disability Research*, *49*(12), 889–904. https://doi.org/10.1111/j.1365-2788.2005.00721.x

Nachshen, J. S., Woodford, L., & Minnes, P. (2003). The Family Stress and Coping Interview for families of individuals with developmental disabilities: a lifespan perspective on family adjustment. *Journal of Intellectual Disability Research*, *47*(4–5), 285–290. https://doi.org/10.1046/j.1365-2788.2003.00490.x

*Neff, K. D., & Faso, D. J. (2015). Self-Compassion and Well-Being in Parents of Children with Autism. *Mindfulness*, *6*(4), 938–947. https://doi.org/10.1007/s12671-014-0359-2

*Nelson-Coffey, S. K., Killingsworth, M., Layous, K., Cole, S. W., & Lyubomirsky, S. (2019). Parenthood Is Associated With Greater Well-Being for Fathers Than Mothers. *Personality and Social Psychology Bulletin*, *45*(9), 1378–1390. https://doi.org/10.1177/0146167219829174

*Nordheim, T., Rustøen, T., Solevåg, A. L., Småstuen, M. C., & Nakstad, B. (2018). Hope in Parents of Very-Low Birth Weight Infants and its Association with Parenting Stress and Quality of Life. *Journal of Pediatric Nursing*, *38*, e53–e58. https://doi.org/10.1016/j.pedn.2017.10.006

Novak, M., & Guest, C. (1989). Application of a Multidimensional Caregiver Burden Inventory1. *The Gerontologist*, *29*(6), 798–803. https://doi.org/10.1093/geront/29.6.798

Östberg, M., Hagekull, B., & Wettergren, S. (1997). A measure of parental stress in mothers with small children: dimensionality, stability and validity. *Scandinavian Journal of Psychology*, *38*(3), 199–208. https://doi.org/10.1111/1467-9450.00028

Paloutzian, R. E., & Ellison, C. W. (1991). Manual for the spiritual well-being scale. Navack: Life Advance.

*Papadopoulos, A., Siafaka, V., Tsapara, A., Tafiadis, D., Kotsis, K., Skapinakis, P., & Tzoufi, M. (2023). Measuring parental stress, illness perceptions, coping and quality of life in families of children newly diagnosed with autism spectrum disorder. *BJPsych Open*, *9*(3), e84. https://doi.org/10.1192/bjo.2023.55

Park, J., Hoffman, L., Marquis, J., Turnbull, A.P., Poston, D., Mannan, H., Wang, M., & Nelson, L. (2003). Toward assessing family outcomes of service delivery: Validation of a family quality of life survey. Journal of Intellectual Disability Research, 47(4/5), 367-384.

Patterson, J. M., & Mccubbin, H. I. (1983). The Impact of Family Life Events and Changes on the Health of a Chronically III Child. *Family Relations*, *32*(2), 255–264. https://doi.org/10.2307/584685

Phetrasuwan, S. (2003). Psychological adjustment in mothers of children with Autism Spectrum Disorder. Chapel Hill, NC: University of North Carolina, Chapel Hill.

Psychiatric Research Unit (1998). WHO (five) well-being index. WHO Collaborating Center for Mental Health, Frederiksborg General Hospital.

*Rai, P., & Rani, U. (2019). Effect of newborn’s admission to intensive care unit on “quality of life” of mother: An Indian perspective. *The Journal of Maternal-Fetal & Neonatal Medicine*, *32*(13), 2188–2193. https://doi.org/10.1080/14767058.2018.1428550

*Rambod, M., Pasyar, N., Mazarei, Z., & Soltanian, M. (2023). The predictive roles of parental stress and intolerance of uncertainty on psychological well-being of parents with a newborn in neonatal intensive care unit: a hierarchical linear regression analysis. *BMC Pediatrics*, *23*(1), 607. https://doi.org/10.1186/s12887-023-04420-4

*Rohde, G., Helseth, S., Mikkelsen, H. T., Skarstein, S., Smastuen, M. C., & Haraldstad, K. (2022). Stress, pain, and work affiliation are strongly associated with health-related quality of life in parents of 14-15-year-old adolescents. *Health and Quality of Life Outcomes*, *20*(1), 1. https://doi.org/10.1186/s12955-021-01913-7

Ryff, C. D. (1989). Happiness is everything, or is it? Explorations on the meaning of psychological well-being. Journal of Personality and Social Psychology, 57(6), 1069–1081. https://doi.org/10.1037/ 0022-3514.57.6.1069

*Salami, S. O. (2007). Multiple role strain and Nigerian working mothers’ psychological well-being: Do self-efficacy, coping strategies and social support make a difference? *European Journal of Social Sciences*, *5*(1), 43–53. Scopus.

*Samadi, S. A., McConkey, R., & Kelly, G. (2013). Enhancing parental well-being and coping through a family-centred short course for Iranian parents of children with an autism spectrum disorder. *Autism*, *17*(1), 27–43. https://doi.org/10.1177/1362361311435156

*Savari, K., Naseri, M., & Savari, Y. (2021). Evaluating the Role of Perceived Stress, Social Support, and Resilience in Predicting the Quality of Life among the Parents of Disabled Children. *International Journal of Disability, Development and Education*. https://doi.org/10.1080/1034912X.2021.1901862

Schmidt, S., Mühlan, H., & Power, M. (2006). The EUROHIS-QOL 8-item index: psychometric results of a cross-cultural field study. *European Journal of Public Health*, *16*(4), 420–428. https://doi.org/10.1093/eurpub/cki155

*Sharda, E. (2022). Parenting Stress and Well-Being Among Foster Parents: The Moderating Effect of Social Support. *Child and Adolescent Social Work Journal*, *39*(5), 547–559. https://doi.org/10.1007/s10560-022-00836-6

*Sharkia, S. A., & Taubman – Ben-Ari, O. (2024). Personal Growth and Life Satisfaction among Arab Mothers After Fertility Treatment – The Role of Stress and Optimism. *Journal of Happiness Studies*, *25*(1–2), 6. https://doi.org/10.1007/s10902-024-00712-3

Silva, L. M. T., & Schalock, M. (2012). Autism Parenting Stress Index: Initial Psychometric Evidence. *Journal of Autism and Developmental Disorders*, *42*(4), 566–574. https://doi.org/10.1007/s10803-011-1274-1

*Silva, N., Bullinger, M., Sommer, R., Rohenkohl, A., Witt, S., & Quitmann, J. (2018). Children’s psychosocial functioning and parents’ quality of life in paediatric short stature: The mediating role of caregiving stress. *Clinical Psychology & Psychotherapy*, *25*(1), e107–e118. https://doi.org/10.1002/cpp.2146

*Skok, A., Harvey, D., & Reddihough, D. (2006). Perceived stress, perceived social support, and wellbeing among mothers of school-aged children with cerebral palsy. *Journal of Intellectual and Developmental Disability*, *31*(1), 53–57. Scopus. https://doi.org/10.1080/13668250600561929

*Staunton, E., Kehoe, C., & Sharkey, L. (2020). Families under pressure: Stress and quality of life in parents of children with an intellectual disability. *Irish Journal of Psychological Medicine*. https://doi.org/10.1017/ipm.2020.4

Stein, R. E. K., & Riessman, C. K. (1980). The Development of an Impact-on-Family Scale: Preliminary Findings. *Medical Care*, *18*(4), 465. https://journals.lww.com/lww-medicalcare/abstract/1980/04000/the_development_of_an_impact_on_family_scale_.10.aspx

*Streisand, R., Mackey, E. R., & Herge, W. (2010). Associations of Parent Coping, Stress, and Well-Being in Mothers of Children with Diabetes: Examination of Data from a National Sample. *Maternal and Child Health Journal*, *14*(4), 612–617. https://doi.org/10.1007/s10995-009-0497-7

*Sun, Y., Cheah, C. S. L., Seo, Y. J., Aquino, A. K., Gürsoy, H., & Wu, L.-W. (2024). All in the family: The complementary protective roles of spousal and other family support for Chinese immigrant mothers’ life satisfaction over time. *Journal of Family Psychology*, *38*(5), 831–837. https://doi.org/10.1037/fam0001212

*Tan, S. A., Pung, P. W., Wu, S. L., Yap, C. C., Jayaraja, A. R., & Chow, B. G. (2024). Parent–child relationship and parents’ psychological well‐being among Malaysian families amid pandemic: The role of stress and gender. *Child & Family Social Work*, *29*(1), 35–47. https://doi.org/10.1111/cfs.13049

Tennant, R., Hiller, L., Fishwick, R., Platt, S., Joseph, S., Weich, S., Parkinson, J., Secker, J., & Stewart-Brown, S. (2007). The Warwick-Edinburgh Mental Well-being Scale (WEMWBS): development and UK validation. *Health and Quality of Life Outcomes*, *5*(1), 63. https://doi.org/10.1186/1477-7525-5-63

The European QoLISSY Group (2013). Quality of life in short stature youth: The QoLISSY questionnaire user's manual. Lengerich: Pabst SciencePublishers.

*Thorsteinsen, K., Heijens, M., Parks‐Stamm, E. J., Froehlich, L., & Martiny, S. E. (2024). The role of gender, stress, and social support in parents’ pandemic well‐being: A cross‐national study. *Family Relations*, fare.13018. https://doi.org/10.1111/fare.13018

*Tien, L. P., Atiqah, N., Vytialingam, N., Rashid, M. A., Kabir, M. S., Farzana, Y., Nirmala, P., Shirin, L., Gupalo, S., & Nazmul, M. H. M. (2022). Stress and Quality of Life Among Fathers of Special Needs Children in Klang Valley. *Journal of Pharmaceutical Negative Results*, *13*, 1010–1018. https://doi.org/10.47750/pnr.2022.13.S06.135

*Vahedparast, H., Khalafi, S., Jahanpour, F., & Bagherzadeh, R. (2022). The Actor–Partner Effects of Parenting Stress on Quality of Life Among Parents of Children with ASD: The Mediating Role of Mental Quality of Life. *Journal of Autism and Developmental Disorders*, *52*(1), 142–149. https://doi.org/10.1007/s10803-021-04919-8

VanderWeele, T. J. (2017). On the promotion of human flourishing. Proceedings of the National Academy of Sciences 114(31), 8148–8156.

Van Katwyk, P. T., Fox, S., Spector, P. E., & Kelloway, E. K. (2000). Using the Job-Related Affective Well-Being Scale (JAWS) to investigate affective responses to work stressors. Journal of Occupational Health Psychology, 5(2), 219- 230. https://doi.org/10.1037/1076-8998.5.2

Varni, J. W., Sherman, S. A., Burwinkle, T. M., Dickinson, P. E., & Dixon, P. (2004). The PedsQL^TM^ Family Impact Module: Preliminary reliability and validity. *Health and Quality of Life Outcomes*, *2*(1), 55. https://doi.org/10.1186/1477-7525-2-55

Vermulst, A., Kroes, G., Meyer, R. D., Nguyen, L., & Veerman, J. W. (2015). *Handleiding OBVL*. Eburon Uitgeverij B.V.

*Wang, H., Hu, X., & Han, Z. R. (2020). Parental stress, involvement, and family quality of life in mothers and fathers of children with autism spectrum disorder in mainland China: A dyadic analysis. *Research in Developmental Disabilities*, *107*. https://doi.org/10.1016/j.ridd.2020.103791

*Wang, Y., Huang, Z., & Kong, F. (2017). Parenting stress and life satisfaction in mothers of children with cerebral palsy: The mediating effect of social support. *Journal of Health Psychology*, *25*(3), 416–425. https://doi.org/10.1177/1359105317739100

Ware, J., Snow, K., Kosinski, M., & Gandek, B. (1992). SF36 health survey: manual and interpretation guide. Lincoln, RI: Quality Metric. *Inc. and The Health Assessment Lab*.

Ware, J. E., Jr., Kosinski, M., & Keller, S. D. (1996). A 12‐Item short‐form health survey: Construction of scales and preliminary tests of reliability and validity. Medical Care, 34(3), 220–233.

Watson, D., Clark, L. A., & Tellegen, A. (1988). Development and validation of brief measures of positive and negative affect: The PANAS scales. *Journal of Personality and Social Psychology*, *54*(6), 1063–1070. https://doi.org/10.1037/0022-3514.54.6.1063

Wiklund, I., Gorkin, L., Pawitan, Y., Schron, E., Schoenberger, J., Jared, L., & Shumaker, S. (1992). Methods for assessing quality of life in the Cardiac Arrhythmia Suppression Trial (CAST). *Quality of Life Research*, *1*, 187–201.

*Witt, W. P., Litzelman, K., Spear, H. A., Wisk, L. E., Levin, N., McManus, B. M., & Palta, M. (2011). Health-related quality of life of mothers of very low birth weight children at the age of five: Results from the newborn lung project statewide cohort study. *Quality of Life Research*, *21*(9), 1565–1576. https://doi.org/10.1007/s11136-011-0069-3blumberg

World Health Organization (1996). Quality of life (WHOQOL)-Brief questionnaire, field trial version. Geneva: WHO.

World Health Organization. (1998). Wellbeing measures in primary health care/the DepCare project: report on a WHO meeting: Stockholm, Sweden, 12–13 February 1998. <https://apps.who.int/iris/handle/10665/349766>

*You, S., Lee, Y., & Kwon, M. (2018). Effect of parenting stress in Korean mothers of children with disabilities on life satisfaction: Moderating effect of intrinsic religious orientation. *Journal of Applied Research in Intellectual Disabilities*, *32*(3), 591–599. https://doi.org/10.1111/jar.12553

Zautra, A. J., Guarnaccia, C. A., & Dohrenwend, B. P. (1986). Measuring small life events. *American Journal of Community Psychology*, *14*(6), 629.

*Zeng, S., Zhao, H., Hu, X., Lee, J. D., Stone-MacDonald, A. K., & Price, Z. W. (2021). Are we on the Same Page: A DyadicAnalysis of Parental Stress, Support, and Family Quality of Life on Raising Children with Autism Spectrum Disorders. *Journal of Developmental and Physical Disabilities*, *33*(4), 599–618. https://doi.org/10.1007/s10882-020-09761-x

*Zhang, W., Liu, T., Zhang, S., & Li, X. (2022). The Mutual Role of Mindful Parenting on Parents’ Subjective Well-being and Young Children’s Emotional Regulation Through Reducing Perceived Parenting Daily Hassles. *Mindfulness*, *13*(11), 2777–2787. https://doi.org/10.1007/s12671-022-01994-5

Zheng, X., Zhu, W., Zhao, H., & Zhang, C. (2015). Employee well-being in organizations: Theoretical model, scale development, and cross-cultural validation. Journal of Organizational Behaviour, 36, 621–644.
